# Modulated expression of human peripheral blood microRNAs from infancy to adulthood and its role in aging

**DOI:** 10.1111/acel.12225

**Published:** 2014-05-06

**Authors:** Chi-Yu Lai, Yen-Tzu Wu, Sung-Liang Yu, Ya-Hui Yu, Su-Yin Lee, Chih-Min Liu, Wu-Shiun Hsieh, Hai-Gwo Hwu, Pau-Chung Chen, Suh-Fang Jeng, Wei J Chen

**Affiliations:** 1 Institute of Epidemiology and Preventive Medicine, College of Public Health, National Taiwan UniversityTaipei, 100, Taiwan; 2 Center of Genomic Medicine, National Taiwan UniversityTaipei, 100, Taiwan; 3 School and Graduate Institute of Physical Therapy, National Taiwan University College of MedicineTaipei, 100, Taiwan; 4 Department of Clinical Laboratory Sciences and Medical Biotechnology, College of Medicine, National Taiwan UniversityTaipei, 100, Taiwan; 5 Department of Psychiatry, College of Medicine and National Taiwan University Hospital, National Taiwan UniversityTaipei, 100, Taiwan; 6 Department of Pediatrics, College of Medicine and National Taiwan University Hospital, National Taiwan UniversityTaipei, 100, Taiwan; 7 Institute of Occupational Medicine and Industrial Hygiene, College of Public Health, National Taiwan UniversityTaipei, 100, Taiwan; 8 Department of Environmental and Occupational Medicine, College of Medicine and National Taiwan University HospitalTaipei 100, Taiwan; 9 Physical Therapy Center, National Taiwan University HospitalTaipei, 100, Taiwan

**Keywords:** aging, development, gene expression profiling, microRNA, network function, peripheral blood

## Abstract

Accumulating evidence suggests a role for microRNAs (miRNAs) in regulating various processes of mammalian postnatal development and aging. To investigate the changes in blood-based miRNA expression from preterm infants to adulthood, we compared 365 miRNA expression profiles in a screening set of preterm infants and adults. Approximately one-third of the miRNAs were constantly expressed from postnatal development to adulthood, another one-third were differentially expressed between preterm infants and adults, and the remaining one-third were not detectable in these two groups. Based on their expression in infants and adults, the miRNAs were categorized into five classes, and six of the seven miRNAs chosen from each class except one with age-constant expression were confirmed in a validation set containing infants, children, and adults. Comparing the chromosomal locations of the different miRNA classes revealed two hot spots: the miRNA cluster on 14q32.31 exhibited age-constant expression, and the one on 9q22.21 exhibited up-regulation in adults. Furthermore, six miRNAs detectable in adults were down-regulated in older adults, and four chosen for individual quantification were verified in the validation set. Analysis of the network functions revealed that differentially regulated miRNAs between infants and adults and miRNAs that decreased during aging shared two network functions: inflammatory disease and inflammatory response. Four expression patterns existed in the 11 miRNAs from infancy to adulthood, with a significant transition in ages 9–20 years. Our results provide an overview on the regulation pattern of blood miRNAs throughout life and the possible biological functions performed by different classes of miRNAs.

## Introduction

Human postnatal development involves sequential changes in an individual’s functions that are influenced by biological determinants and environmental experiences, starting from the neonatal period to infancy, childhood, adolescence, and maturity (Martini & Nath, 2009). Many growth hormones function as key regulators during postnatal development, such as pituitary growth hormone and the sex hormones testosterone and estrogen. The cross talk among hormones and signaling cascades in regulating specific genes is likely to determine cell growth, differentiation, metabolism, and cytoskeletal changes (Piwien-Pilipuk *et al*., 2002).

Accumulating evidence suggests a role for microRNAs (miRNAs), endogenous noncoding single-stranded RNAs of approximately 22 nucleotides, in regulating various processes of mammalian postnatal development (Alvarez-Garcia & Miska, 2005; Sayed & Abdellatif, 2011) and aging (Noren Hooten *et al*., 2010; Elsharawy *et al*., 2012). The finding of aberrant expression in several blood-based miRNAs has also raised the possibility that these miRNAs serve as novel diagnostic markers for a variety of disorders (Kosaka *et al*., 2010; Lai *et al*., 2011). For example, miRNA expression profiling in the peripheral blood of healthy humans has indicated that many predicted target genes of miRNAs in peripheral blood mononuclear cells are required for embryonic development (Liang *et al*., 2007), and some miRNAs may serve as biomarkers for development and age-related diseases as well as the transition between development and aging (Noren Hooten *et al*., 2010; Somel *et al*., 2010; Beveridge *et al*., 2014).

The distinction between the neonatal period and adulthood varies considerably between multiple cellular regulatory processes, which are likely to be regulated by miRNAs (Alvarez-Garcia & Miska, 2005). A study of miRNA profiling in nematodes showed that approximately one-third of miRNAs exhibit changes in expression during adulthood (Ibanez-Ventoso *et al*., 2006). When reaching maturation, aging begins with a process of various stimuli, such as telomere dysfunction and oxidative stress, which can induce irreversible cell growth arrest, called cellular senescence (Campisi & d’Adda di Fagagna, 2007). Several miRNAs promote aging by modulating their targets to drive cell senescence and aging in different tissues or organs, such as human epididymis, skeletal muscle, brain, and peripheral blood (Lukiw, 2007; Drummond *et al*., 2008; Noren Hooten *et al*., 2010; Somel *et al*., 2010; Zhang *et al*., 2010). Furthermore, the majority of these age-regulated miRNAs are down-regulated in old humans (Ibanez-Ventoso *et al*., 2006; Noren Hooten *et al*., 2010; Beveridge *et al*., 2014).

To date, little is known about the changes in the blood-based expression of human miRNAs before and after adulthood, and few studies have investigated the link between developmental regulation and expression changes in aging (Somel *et al*., 2010). The aim of this study was to investigate whether peripheral blood miRNAs are differentially expressed from preterm infants to adults. We compared the expression levels of 365 miRNAs between preterm infants (*n* = 30, with gestational age ranging from 24 to 33 weeks) and adults (*n* = 60, with age ranging from 21 to 61 years) as well as between young and middle-aged adults and then explored the possible biological functions of these miRNAs. Finally, we validated the results of 11 miRNAs in a validation set of independent samples of preterm infants (*n* = 22, with gestational age ranging from 24 to 35 weeks) and adults (*n* = 68, with age ranging from 21 to 65 years). In addition, we included children (*n* = 66, with age ranging from 9 to 10 years) in the validation set to add the information on the transition in miRNA expression from infancy to adulthood.

## Results

**Figure 1:**
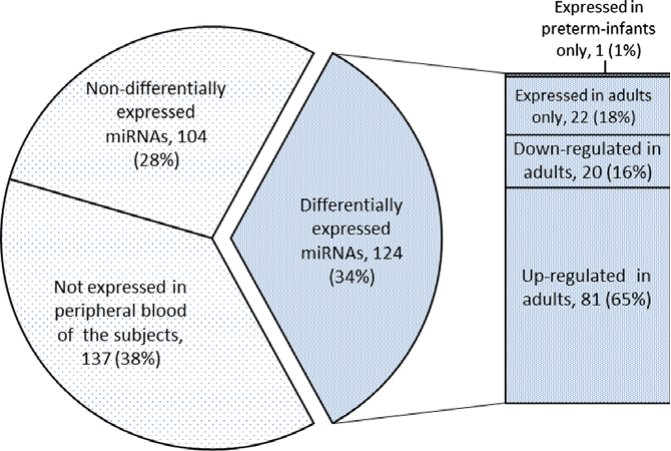
Peripheral blood miRNA expression patterns between the preterm infants (*n* = 30) and adults subjects (*n* = 60). In the pie chart, a total of 365 miRNAs were divided into expressed (228 miRNAs) and nonexpressed miRNAs (137 miRNAs) in the peripheral blood samples. The expressed miRNAs included nondifferentially expressed miRNAs (104 miRNAs) and differentially expressed miRNAs (101 miRNAs). The bar chart of differentially expressed miRNAs contains two major groups, the miRNAs expressed in one group only (23 miRNAs), and the miRNAs expressed in both groups but up- (81 miRNAs) or down-regulated in the adults (20 miRNAs).

The age distribution and gender distribution between the screening set and the validation set of study participants were similar (Table S1). Figure 1 depicts the lifespan pattern of expression of 365 miRNAs in the screening set, comparing preterm infants with adults. Among the 365 miRNAs, 137 (38%) were detectable neither in the adult nor in the preterm infant group, 104 (28%) were nondifferentially expressed between preterm infants and adults, and 124 (34%) were differentially expressed between preterm infants and adults. Among the 124 differentially expressed miRNAs, 1 (1%) was expressed in preterm infants only, 22 (18%) were expressed in adults only, 20 (16%) were down-regulated in adults, and 81 (65%) were up-regulated in adults.

### Classification of miRNAs based on age-related expression

The relationship between miRNA expressivity and age was used to classify the miRNAs into five classes: age-constant expression, age-limited expression-preterm infants only, age-limited expression-adults only, age-related down-regulated in adults, and age-related up-regulated in adults (Table 1). Among the miRNAs that were detectable in adults, six exhibited decreasing expression from young adulthood to middle-aged adulthood. The top 5% differentially expressed miRNAs (*n* = 7) of those classes that showed age-limited or age-related expression and the miRNAs with aging-diminished expression that were chosen for individual quantification (*n* = 4) in the validation set of participants are also listed in Table 1.

**Table 1:** Expression-based classification of 228 miRNAs that had detectable expression either in preterm infants (*n* = 30) or adults (*n* = 60) and the top-ranked associated network functions as revealed using Ingenuity Pathway Analysis software

Class of miRNAs	No. miRNAs	Network Score†	No. focus miRNAs‡	Annotated function categories	miRNAs chosen for validation§
Age-constant expression	104	54	24	Hereditary disorder Skeletal and muscular disorders Developmental disorder	Not applicable
Age-limited expression-preterm infants only	1	4	1	Endocrine system disorders Reproductive system disease	hsa-miR-325
Age-limited expression-adults only	22	34	13	Reproductive system disease Cell morphology Cellular function and maintenance	hsa-miR-1
Age-related down-regulation in adults	20	46	16	Inflammatory disease Inflammatory response Renal inflammation	hsa-miR-486
Age-related up-regulation in adults	81	76	29	Connective tissue disorders Inflammatory disease Inflammatory response	hsa-26a, hsa-miR-24, hsa-miR-26b, and hsa-miR-142-3p
Aging-diminished expression	6	18	6	Connective tissue disorders Inflammatory disease Inflammatory response	hsa-let-7a, hsa-miR-30e-5p, hsa-miR-410, and hsa-miR-107

† Network Score = −log (*P*-value of Fisher’s exact test).

‡ Focus miRNAs are the seed miRNAs that interact with other molecules of the network in the Ingenuity Knowledge Base of Ingenuity Pathway Analysis (version 9.0, release in June 2012, Ingenuity® Systems).

§ For those classes that showed age-limited or age-related expression, the top 5% differentially expressed miRNAs were chosen; for the miRNAs with aging-diminished expression, those showing either significant correlation with aging or reported to have biological relevance with aging were chosen (see the text for more detail).

The miRNAs of each class were then subjected to the knowledge-based software Ingenuity Pathway Analysis (IPA) to search for the associated network with the highest network score. The results and annotated function categories are summarized in Table 1. Interestingly, there was much overlap between the annotated functions of the two classes of miRNAs with age-related modulation, including inflammatory disease and inflammatory response.

For each class of miRNAs, individual names and the corresponding chromosomal locations and ordinates are listed in Table S2 (age-constant expression), Table S3 (age-limited expression, including preterm infants only and adults only), Table S4 (down-regulated in adults), and Table S5 (up-regulated in adults).

### Expression profiling for differentially expressed miRNAs

**Figure 2:**
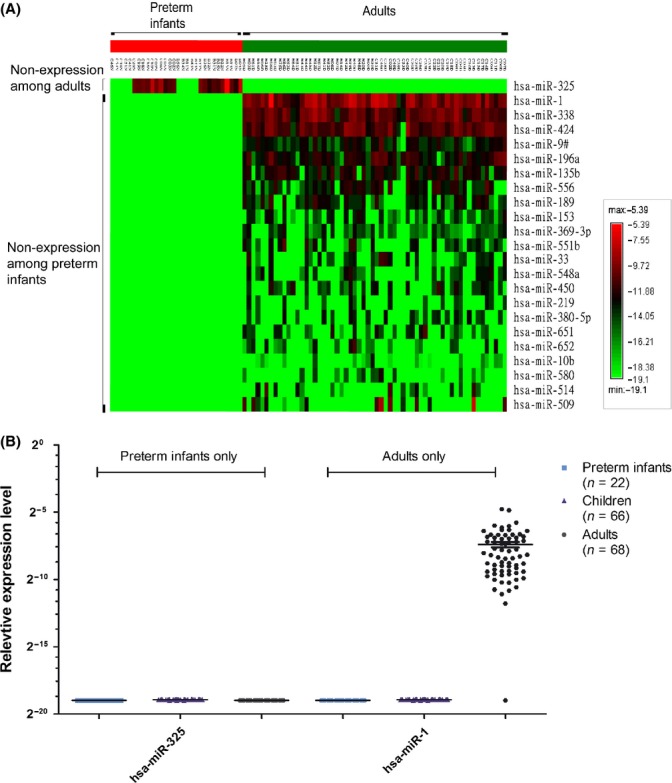
Expression patterns of miRNAs with age-limited expression. (A) Among the 23 miRNAs that were expressed in adults or infants only, miR-325 was expressed in preterm infants only, and the other 22 miRNAs were expressed in adults only. (B) For the two miRNAs chosen from the class of age-limited expression for validation, hsa-miR-325 showed no expressions in all samples and miR-1 showed expression in the adults only.

For miRNAs with differential expression between preterm infants and adults, we applied a heatmap to illustrate their expression profiles. For the 23 miRNAs with age-limited expression (Fig. 2A), miR-325 was exclusively expressed in two-thirds of preterm infants. To explore the function of miR-325 in preterm infants, we compared the perinatal characteristics of preterm infants with miR-325 expression (*n* = 20) with those of preterm infants without miR-325 expression (*n* = 10). We found that preterm infants with miR-325 expression had a lower probability (10%) of suffering from periventricular leukomalacia than those without miR-325 expression (50%) (*P* = 0.015). The association was also demonstrated in the validation set of preterm infants (*n* = 22), with all of them showing nonexpression of miR-325 (Fig 2B) and 86.4% of them suffering from periventricular leukomalacia. The other 22 miRNAs were expressed in at least one-third of the adults. Of those, miR-1 was highly expressed in all adults (Fig. 2A). When examined in the validation set, miR-1 exhibited similar expression pattern, showing nonexpression in both the preterm infants and children but detectable expression in the adults (Fig. 2B).

**Figure 3:**
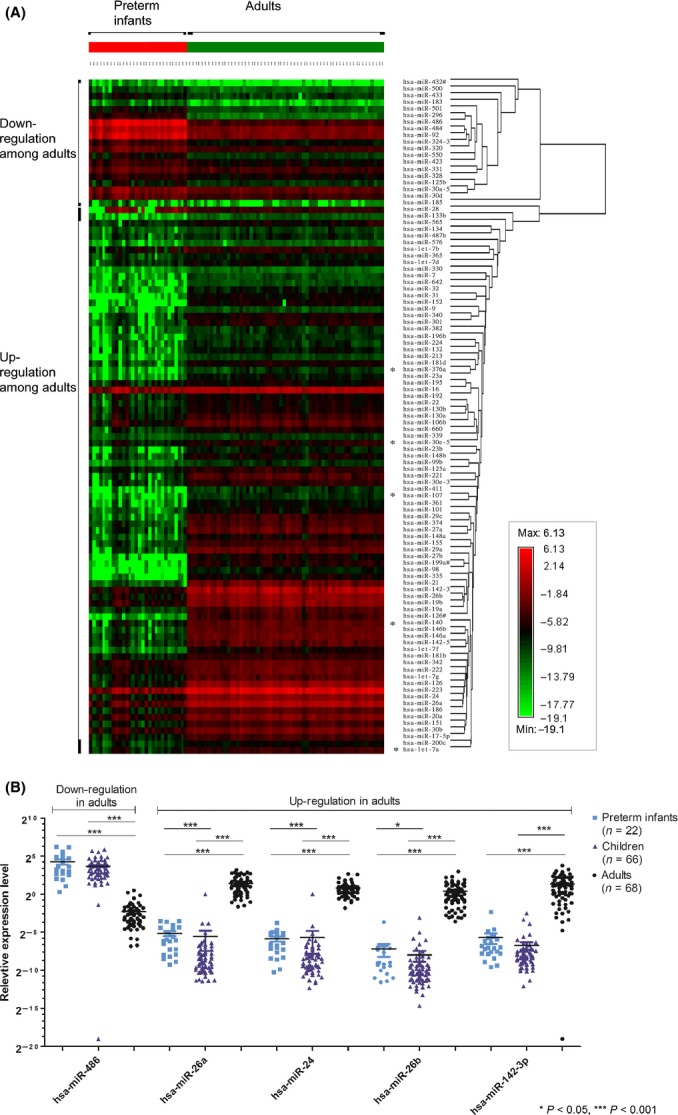
Expression patterns of miRNAs with age-related modulations in expression. (A) Among the 101 differentially expressed miRNAs between adults and infants, 20 miRNAs were down-regulated and the remaining 81 were up-regulated in the adults (*P* < 0.00024 with Bonferroni correction). (B) For the miRNA (hsa-miR-486) chosen from the class of down-regulation in adults for validation, its expression pattern was confirmed by showing down-regulation (*P* < 0.0001) in the adults as compared to the preterm infants and the children, with the latter two showing no difference in their expression levels. For the four miRNAs (miR-26a, miR-24, miR-26b, and miR-142-3p) chosen from the class of up-regulation in adults, their expression patterns were also confirmed by showing up-regulation (*P* < 0.0001) in the adults as compared to the preterm infants and the children, with three of them (miR-26a, miR-26b, and miR-24) being down-regulated from infancy to childhood.

For miRNAs with age-related modulation in expression, the contrast between the 20 down-regulated and 81 up-regulated miRNAs in adults is displayed in Fig. 3A. For the miRNA (hsa-miR-486) chosen from the class of down-regulation in adults for validation, its expression pattern was confirmed by showing down-regulation (*P* < 0.0001) in the adults as compared to the preterm infants and the children, with the latter two showing no difference in their expression levels (Fig. 3B). For the four miRNAs (miR-26a, miR-24, miR-26b, and miR-142-3p) chosen from the class of up-regulation in adults, their expression patterns were also confirmed by showing up-regulation (*P* < 0.0001) in the adults as compared to the preterm infants and the children, with three of them (miR-26a, miR-26b, and miR-24) being down-regulated from infancy to childhood (Fig. 3B).

### Chromosomal locations of miRNA genes

We identified 264 chromosomal locations for the 228 miRNAs with detectable expression in this study (each miRNA may have more than one location). For the five expression-based classes of miRNAs, the distributions of their chromosomal locations are shown in Table S6. Some classes of miRNAs appeared to concentrate on certain chromosomes. For example, the proportion of miRNAs with age-constant expression located on chromosome 14 (*n* = 23, 20.4%) was higher than the proportion of all detectable miRNAs located on chromosome 14 (34, 12.9%); the proportion of age-limited miRNAs located on the X chromosome (9, 29.0%) was higher than the proportion of all detectable miRNAs on the X chromosome (33, 12.5%); and the proportions of adult-up-regulated miRNAs located on chromosome 9 (14, 14.6%) and chromosome 19 (10, 10.4%) were higher than the corresponding proportions of all detectable miRNAs (18, 6.8% and 13, 4.9%, respectively).

We then further examined the individual miRNA loci on these four class-associated chromosomes (Table S7) to identify any locus that had a cluster of more than five miRNAs belonging to the same class. As shown in Table 2, there were two such chromosomal hot spots: 14q32.31 and 9q22.32. Of the 23 miRNAs located on 14q32.31, 16 (69.6%) belonged to the class of age-constant expression, which was higher than the average proportion of 45.6%. In contrast, six miRNAs were located on 9q22.32, all of which were up-regulated in adults.

**Table 2:** Expression-based classification of miRNAs that had detectable expression either in preterm infants (*n* = 30) or adults (*n* = 60) and their chromosomal regions

Expression-based classification of miRNAs	Genome-wide	Chromosomal hot spot	
		14q32.31	9q22.32
Total with detectable expression	228	23	6
Age-constant expression	104 (45.6%)	16 (69.6%)	0 (0%)
Age-limited expression			
Expressed in preterm infants only	1 (0.4%)	0 (0%)	0 (0%)
Expressed in adults only	22 (9.7%)	2 (8.7%)	0 (0%)
Age-related modulation in expression			
Down-regulated in adults	20 (8.8%)	0 (0%)	0 (0%)
Up-regulated in adults	81 (35.5%)	5 (21.7%)	6 (100%)

The miRNAs of each chromosomal hot spot were then subjected to the microRNA Target Filter analysis implemented in the IPA to search for the associated predicted target genes with the criteria of ‘high confidence’ or ‘experimentally observed’. For the 16 miRNAs with age-constant expression located on 14q32.31, 15 miRNAs have predicted miR-target genes annotated in the IPA database, with a total of 3209 target genes being identified. Among these genes, 666 were commonly targeted by at least two miRNAs. For the six miRNAs that were up-regulated in adults located on 9q22, two miRNAs have predicted miR-target genes, with a total of 1793 target genes being identified. Among these genes, 130 were commonly targeted by these two miRNAs.

Two sets of target genes were then subjected to canonical pathway analyses using the IPA (Table S8). Of note, for the miR-target genes of the miRNAs that were clustered on 14q32.31, four of the five associated canonical pathways belong to organismal growth and development pathway category. For the miR-target genes of the miRNAs that were clustered on 9q22, four of the five associated canonical pathways belong to cancer pathway category (Table S8). The target genes derived from the top five associated canonical pathways for the age-constant expression miRNAs clustered on 14q32.31 are displayed in Table S9, and those for the age-related up-regulation expression in adults miRNAs clustered on 9q22.32 are displayed in Table S10.

### Postadulthood diminishing expression with age

When all of the adults were divided into young (≤ 35 years, *n* = 32) and middle-aged (> 35 years, *n* = 28) adults, six miRNAs (hsa-let-7a, miR-30e-5p, miR-107, miR-140, miR-376a, and miR-410) exhibited decreasing expression from young adulthood to middle-aged adulthood according to Benjamini and Hochberg (BH) false discovery rate correction (Table 3). These six miRNAs were then subjected to correlation analyses, and three (hsa-let-7a, miR-410, and miR-107) of them were significantly decreased with increasing age (*r* = −0.34 to −0.40, *P* < 0.0083, with Bonferroni correction) (Fig. S2). Furthermore, hsa-let-7a and miR-107 were negatively correlated with age (*r* = −0.40, *P* < 0.0083, with Bonferroni correction) after adjustment for gender. Their annotated network functions, as shown in Table 1, included connective tissue disorders, inflammatory disease, and inflammatory response.

**Table 3:** The miRNAs (*n* = 6) that had diminishing expression with age, from young (≤ 35 years old) to old adults (> 35 years old), and their chromosomal regions

miRNA name	Chromosomal region	Chromosomal coordinates¶	Screening set (*N*: young = 32, middle-aged = 28)		Validation set (*N*: young = 34, middle-aged = 34)	
			Fold change†	*P*^-^value‡	Fold change†	*P*^-^value§
hsa-let-7a	9q22.32	9: 96938239-96938318 [+]	−1.6	0.0004	−1.6	0.007
	11q24.1	11: 122017230-122017301 [−]				
	22q13.31	22: 46508629-46508702 [+]				
hsa-miR-30e-5p	1p34.2	1: 41220027-41220118 [+]	−1.7	0.003	−2.1	0.005
hsa-miR-140	16q22.1	16: 69966984-69967083 [+]	−1.4	0.003	NA	NA
hsa-miR-410	14q32.31	14: 101532249-101532328 [+]	−2.1	0.003	−2.4	0.003
hsa-miR-376a	14q32.31	14: 101507119-101507186 [+]	−1.9	0.004	NA	NA
	14q32.31	14: 101506406-101506485 [+]				
hsa-miR-107	10q23.31	10: 91352504-91352584 [−]	−1.6	0.005	−1.5	0.02

NA, not available (the miRNA was not included for individual quantification in the validation set).

† Fold change = 2^−(ΔCt, young adults)^/2^−(ΔCt, middle-aged adults))^; a negative value means decreasing expression with age.

‡ Crude *P*-value based on Wilcoxon rank-sum test; only those miRNAs that remained significant with BH false discovery correction at the 20% level (i.e., a crude *P*-value of < 0.006) were listed here.

§ Based on Wilcoxon rank-sum test.

¶ [+]: forward strand; [−]: reverse strand.

Four (hsa-let-7a, miR-30e-5p, miR-410, and miR-107) of the six miRNAs of the class of aging-diminished expression were chosen for validation in meeting one of the following criteria: (i) the expression levels of the miRNAs were significantly correlated with aging after Bonferroni correction (hsa-let-7a, miR-410, and miR-107 as shown in Fig. S2); or (ii) the miRNAs were reported to have biological relevance with aging (hsa-let-7a, miR-30e-5p, and miR-107). In the validation set, all of the four miRNAs exhibited significantly differential expression between the young (≤ 35 years, *n* = 34) and the middle-aged (> 35 years, *n* = 34) adults (Table 3). Furthermore, except miR-107, the correlation coefficients with age for the other three miRNAs (hsa-let-7a, miR-30e-5p, and miR-410) ranged from −0.27 to −0.36 (*P* = 0.02 to 0.003) (Fig S3). After adjustment for gender, both miR-30e-5p and miR-410 remained significantly correlated with age (*r* = −0.33 to −0.36, *P* < 0.0083, with Bonferroni correction).

### The transition of miRNA expressions from infancy to adulthood

**Figure 4:**
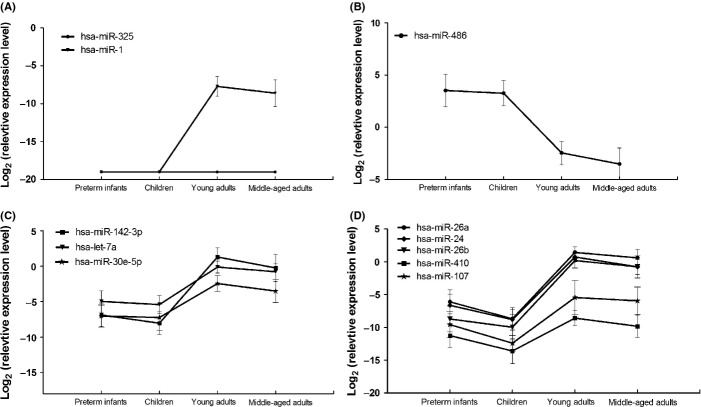
The age-related changes in the expression of the 11 miRNAs chosen for qPCR. The expression level of each miRNA was presented using the mean -∆Ct and the standard deviation. (A) hsa-miR-1 was expressed in adulthood only and was not associated with aging. (B) miR-486 was constantly expressed from infancy and childhood, down-regulated in young adulthood, and then diminished with aging. (C) miR-142-3p, let-7a, and miR-30e-5p were constantly expressed from infancy and childhood, up-regulated in young adulthood, and then showed expression diminishing with aging. (D) miR-26a, miR-24, miR-26b, miR-410, and miR-107 were down-regulated from infancy to children, up-regulated in young adulthood, and then showed expression diminishing with aging.

As the validation set included children, it is of interest to examine the age-related changes in the expression of the 11 miRNAs chosen for qPCR. Four patterns in the transition of miRNAs’ expression from infancy to adulthood were identified. First, hsa-miR-1 was expressed in adulthood only and was not associated with aging (Fig. 4A). Second, miR-486 was constantly expressed from infancy and childhood, down-regulated in young adulthood, and then diminished with aging (Fig. 4B). Third, miR-142-3p, let-7a, and miR-30e-5p were constantly expressed from infancy and childhood, up-regulated in young adulthood, and then showed expression diminishing with aging (Fig. 4C). Fourth, miR-26a, miR-24, miR-26b, miR-410, and miR-107 were down-regulated from infancy to children, up-regulated in young adulthood, and then showed expression diminishing with aging (Fig. 4D).

### The networks of regulatory interactions

**Figure 5:**
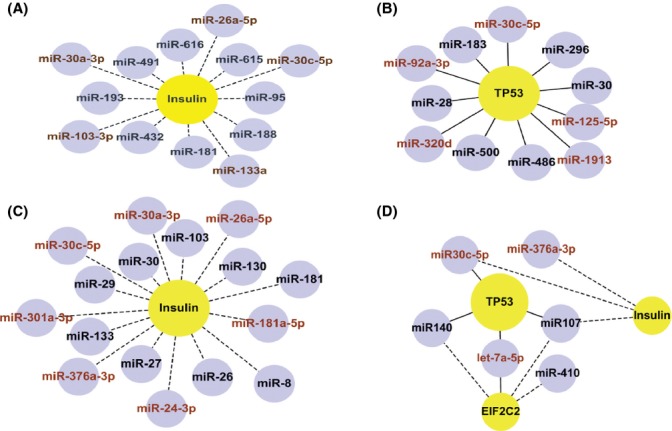
The network of regulatory interactions identified in different classes of miRNAs. Only part of the full network (shown in Figure S1) with the highest Network Score is shown here. The represented molecules are the hub molecules (in yellow circles) that showed an interaction with specific miRNAs (in small blue circles). The networks are illustrated separately for the miRNA classes of (A) age-constant expression, (B) expressed in both groups but down-regulated in adults, (C) expressed in both groups but up-regulated in adults, and (D) decreased expression from young to old adults. The indirect interactions are represented by dashed lines, and the direct interactions are represented by solid lines. Further, the miRNA nodes with the name in brown color represent those miRNAs with the same seed sequence. The network was drawn using Cytoscape software (version 2.8.3; http://www.cytoscape.org/).

Among the associated networks of different classes of miRNAs, two hub molecules, that is, nodes that have many links in a network, were revealed: the insulin protein complex and tumor suppressor p53. The insulin protein complex appeared to interact with miRNAs with age-constant expression (Fig. 5A) and with miRNAs that were up-regulated in adults (Fig. 5C). p53 appeared to interact with miRNAs that were down-regulated in adults (Fig. 5B) and aging-diminished miRNAs (Fig. 5D).

## Discussion

In this study, we compared the expression levels of peripheral blood miRNAs from preterm infancy to adulthood. Five major findings emerged. First, approximately one-third of the miRNAs were constantly expressed from postnatal development to adulthood, another one-third were differentially expressed between preterm infants and adults, and the remaining one-third were not detectable in these two sample populations. Based on their expression in infants and adults, the miRNAs were categorized into five classes. Six (miR-1, miR-486, miR-26a, miR-24, miR-26b, and miR-142-3p) of seven top 5% differentially expressed miRNAs in the classes with age-limited or age-related expression were confirmed in a validation set using qPCR. Second, when comparing the distribution of the chromosomal locations of the different classes of miRNAs, we found two chromosomal hot spots, with the miRNA cluster on 14q32.31 exhibiting age-constant expression and the one on 9q22.21 exhibiting up-regulation in adults. Pathway analyses revealed that the miRNA cluster on 14q32.31 was mostly associated with the category of organismal growth and development pathway, and the miRNA cluster on 9q22 associated with cancer pathway. Third, six miRNAs that were detectable in adults diminished with aging. All of the four miRNAs (hsa-let-7a, miR-30e-5p, miR-410, and miR-107) chosen for validation showed similar age-diminishing trend. Fourth, based on the expressions of the 11 miRNAs chosen for validation, four patterns in the transition of miRNAs’ expression from infancy to adulthood were identified. Finally, on the basis of the network functions analyses, the miRNAs with age-related modulation in expression as well as the aging-diminished miRNAs all had network functions in both inflammatory disease and inflammatory response. Our results provide an overview on the regulatory pattern of peripheral blood miRNAs throughout life and the possible biological functions performed by different classes of miRNAs.

The miRNAs with age-constant expression are likely to serve as regulators of essential physiologic processes, such as essential functions during animal development, regulation of cellular differentiation, and helping establish tissue-specific gene expression programs (Chang & Mendell, 2007). Subsequent IPA-based analyses of the associated network functions further identified the insulin protein complex as the hub molecule for interactions among the miRNAs of this class (discussed later). Thus, aberrant expression of these miRNAs could result in human disease (Alvarez-Garcia & Miska, 2005; Chang & Mendell, 2007).

Our finding that approximately one-third of miRNAs were modulated from preterm infants to adulthood is consistent with previous findings in model organisms, for example, *Caenorhabditis elegans* (Ibanez-Ventoso *et al*., 2006), and human postmortem brain tissue (Somel *et al*., 2010; Beveridge *et al*., 2014). This supports the postulation that miRNAs serve as key postnatal development- and aging-related regulators across species and tissues (Noren Hooten *et al*., 2010). Furthermore, the transition of miRNAs’ expression levels in peripheral blood at around 9 to 20 years of age may signal the beginning of systemic changes associated with puberty. It is noteworthy that our expression classes of miRNAs in peripheral blood derived from the array-based analyses as well as the transition from infancy to adulthood from the individual qPCR quantification are part of the patterns identified from the miRNAs expression changes with age in the brain of humans and rhesus macaques (Somel *et al*., 2010).

For the single miRNA that was expressed in preterm infants only, miR-325, its absence of expression was associated with periventricular leukomalacia, which is a major precursor for neurological and intellectual impairment (e.g., cerebral palsy) in later life (Rezaie & Dean, 2002). A recent rat study on lipopolysaccharide-induced periventricular leukomalacia revealed the dysregulation of two miRNAs (mo-miR-451 and mo-miR-200b) in the rat brain tissue (Guo *et al*., 2013). Hence, our finding about the dysregulation of miR-325 in preterm infants, despite the limited significance level due to small sample size, provides a useful clue for the development of biomarker for periventricular leukomalacia that may lead to its prevention or treatment. Regarding the turning-off in the expression of miR-325 in adults, previous studies have also found that miR-325 is undetectable in the peripheral blood of adults (Liang *et al*., 2007). Intriguingly, a recent study examining the expression of miR-325 in the placental tissues of pregnant females reported that it was down-regulated in preeclamptic patients compared with patients with noncomplicated pregnancies (Lazar *et al*., 2012). Whether this indicates that miR-325 is turned on in pregnant adults or that insufficient expression leads to pregnancy-related hypertensive disorders warrants further investigation.

Among the miRNAs with age-limited expression, the majority of them were expressed in adults only, with miR-1 being the most highly expressed in the peripheral blood of all adults. Evidence suggests that miR-1 is a highly conserved miRNA with common muscle-specific expression during embryonic and fetal development as well as during adulthood in flies, zebrafish, chicken, mice, and humans (Chen *et al*., 2006; Ibanez-Ventoso *et al*., 2006; Chang & Mendell, 2007; Liang *et al*., 2007; Koutsoulidou *et al*., 2011). Nevertheless, the expression of miR-1 is increased in the bloodstream of patients with myocardial infarction (Cheng *et al*., 2010). Taken together, these findings imply that adult muscle tissue might regularly release a certain level of miR-1 into the blood for baseline function, and elevated expression of miR-1 might serve as a biomarker for muscle cell injury, such as acute myocardial infarction.

When young adults were compared with middle-aged adults, six miRNAs exhibited diminishing expression with age. One of the miRNAs, miR-107, was also found in a previous study (Noren Hooten *et al*., 2010) to be down-regulated in the peripheral blood of old people, and three of the miRNAs (hsa-let-7a, miR-30e-5p, and miR-107) have been implicated in human cancers (Moncini *et al*., 2011; Thornton & Gregory, 2012). Thus, reduced levels of both the miR-30 family and miR-107 in old adults seem to protect these individuals from malignant mesothelioma and neuroblastoma, whereas reduced levels of the let-7 family in old adults seem to promote tumor progression. This may help account for the epidemiological findings that neuroblastoma is the most common solid cancer in childhood, whereas the incidence of lung cancer, gastric cancer, and breast cancer increase with aging.

Two chromosomal hot spots for miRNA genes, 14q32.31 and 9q22.32, were identified in this study. The results of the IPA-based pathway analyses imply that their functional relevance was organismal growth and development pathway (14q32.31) or cancer pathway (9q22.32), respectively. Most (~70%) of the miRNAs that clustered on 14q32.31, a conserved genomic region for the imprinted *DOLK1-DIO3* domain (Benetatos *et al*., 2013), had age-constant expression. This genomic region contains paternally expressed imprinted genes and maternally expressed miRNAs, with many of these miRNAs differentially expressed in several pathologic processes and various cancers (Benetatos *et al*., 2013). For example, dysregulation of the miRNAs belonging to this genomic region is correlated with gastrointestinal stromal tumors and a high risk of neuroblastoma (Haller *et al*., 2010; Gattolliat *et al*., 2011). The six miRNAs clustered on 9q22.32 were expressed in both preterm infants and adults but were up-regulated in adults, and less information is available about them. Among these miRNAs, the expression of the miR-23/27/24 cluster is involved in angiogenesis and endothelial apoptosis in cardiac ischemia and retinal vascular development (Bang *et al*., 2012).

One prominent hub in the IPA-derived associated network functions was the insulin protein complex, including the insulin hormone and insulin-like growth factor proteins. This hub interacted with two classes of miRNAs, one with age-constant expression and the other with age-related modulation. The interactions between the insulin protein complex and miRNAs with age-constant expression may involve many important anabolic processes, including carbohydrate, lipid, and protein metabolism in muscles, adipose tissue, liver, and other target tissues throughout life (Dimitriadis *et al*., 2011). Furthermore, the level of the insulin protein complex increases with growth during childhood (Lofqvist *et al*., 2005) as well as during puberty (Lofqvist *et al*., 2005; Kail & Barnfield, 2012). Thus, the insulin protein complex might be involved in the regulation of cellular stress response pathways, which also become more active with growth (Chen *et al*., 2010).

A second hub molecule, p53, was involved with miRNAs that were down-regulated from preterm infancy to adulthood as well as with aging-diminished miRNAs. This inverse correlation of miRNAs with p53 might account for the prevention of tumorigenesis by the down-regulation of these miRNAs in adulthood and during aging (Brosh *et al*., 2008; Elsharawy *et al*., 2012). For example, the down-regulation of miR-30 family and miR-107 can up-regulated p53 expression in human cell lines (Li *et al*., 2010), and overexpression of miR-125b represses the endogenous level of p53 protein and suppresses apoptosis in human neuroblastoma cells and human lung fibroblast cells (Le *et al*., 2009). Thus, during development and aging, the shortening of telomeres might induce p53-mediated senescence activity through the modulation of miRNAs in the p53 network (Hermeking, 2012). Inflammatory disease and the inflammatory response were the common network functions associated with miRNAs that showed age-related modulation (either up- or down-regulation in adults) and aging-related diminution. These miRNAs might be involved in the immune response and in the regulation of immunosenescence in humans (Liston *et al*., 2012; Seeger *et al*., 2013). These findings are similar to those of a meta-analysis of age-related gene expression profiles using datasets from mice, rats, and humans (de Magalhaes *et al*., 2009).

Our study has some limitations. First, the study samples of preterm infants in both the screening and the validation set, and the adults in the screening set consisted of heterogeneous groups: preterm infants with and without bronchopulmonary dysplasia as well as adult patients with schizophrenia and normal controls. There are only limited existing data to evaluate the impact of including such heterogeneous subgroups of participants in the analysis on the findings. For example, among the 23 miRNAs with age-limited expression and 101 differentially expressed miRNAs, one (miR-652) was up-regulated in schizophrenia (Lai *et al*., 2011), and three others were either down-regulated (miR-152) or up-regulated (miR-133b and miR-7) in bronchopulmonary dysplasia (Wu *et al*., 2013), all in peripheral blood. Meanwhile, among the 101 differentially expressed miRNAs, 12 (Tables S4 and S5) were found to be down-regulated in the postmortem brain tissue of patients with schizophrenia (Gardiner *et al*., 2012). While it cannot be totally excluded that the age-related or age-limited changes are due to disease status per se, the changes in the expression of these miRNAs with age seem not to be simply due to the disease status for the following two considerations: (a) the heat maps of the differentially expressed miRNAs (Figs 2 and 3) show that the age effect is much stronger than disease effect; and (b) in view of the direction of gene expression changes, except miR-652, miR-28-5p, miR-133b, and miR-7, the age effect on the expressions of other 12 disease-related miRNAs would be under-estimated rather than over-estimated. For example, miR-181a was found to be down-regulated in patients with schizophrenia but up-regulated in adults compared to preterm infants (Gardiner *et al*., 2012). Second, little is known about miRNA dysregulation in preterm birth per se. The preterm infants we recruited in this study might have had some development-related dysregulation in their miRNAs, such as miRNAs involved in neuronal and muscle development, hematopoiesis, and organogenesis (Sayed & Abdellatif, 2011). Some of the differentially expressed miRNAs between preterm infants and adults might also have resulted from preterm birth-related miRNA changes. Finally, although the sample size of this study (*n* > 30 in each group) was estimated to have sufficient power (> 80%) in detecting a difference with an effect size of ≥ 1.1 using the simple two-group comparison, the heterogeneity of our sample could lower the power to get overly strong evidence.

In conclusion, our results indicate that peripheral blood miRNAs exhibit changes in expression and regulatory interaction networks during postnatal development and aging. These results will help us better understand the roles of peripheral blood miRNA expression changes in relation to postnatal development and aging. The potential of these peripheral blood miRNAs as biomarkers for human development and aging warrants further investigation.

## Methods

### Participants

The participants of this study included two independent sets: a screening set and a validation set. The screening set consisted of preterm infants (*n* = 30, mean gestational age = 29.1 weeks) and adults (*n* = 60, mean age = 37 years), and the validation set consisted of independent samples of preterm infants (*n* = 22, mean gestational age = 28.9 weeks), children (*n* = 66, mean age = 9.1 years), and adults (*n* = 68, mean age = 36.9 years). For the preterm infants, we enrolled very-low-birth-weight preterm infants who were admitted to the neonatal intensive care unit at the National Taiwan University Hospital and Mackey Memorial Hospital in Taipei, Taiwan, from September 2008 to December 2009, as described in more detail in a previous study (Wu *et al*., 2013). Briefly, the inclusion criteria included gestational age ≤ 33 weeks, birth weight < 1500 g, absence of congenital and chromosomal abnormalities, and infants who were born in or admitted to the neonatal intensive care unit within 7 days of birth. Subjects were excluded if their parents were of non-Taiwanese ethnicity, the mother’s age was < 18 years, or the mother had a history of substance abuse or a psychiatric disorder. The first 30 preterm infants (15 with bronchopulmonary dysplasia and 15 without bronchopulmonary dysplasia) were used in the screening set, and another 22 preterm infants (12 with bronchopulmonary dysplasia and 10 without bronchopulmonary dysplasia) were used in the validation set.

For the adults, we enrolled 30 patients who met the Diagnostic and Statistical Manual of Mental Disorders, Fourth Edition criteria for schizophrenia at National Taiwan University Hospital, along with 30 age- and gender-matched, unrelated healthy controls who were recruited from a pool of staff, graduate students, and community volunteers from December 2007 to September 2009, as described in another study (Lai *et al*., 2011). Individuals with severe neurological abnormalities, emotional disorders, mental retardation, or prominent substance abuse problems were excluded. Another 68 healthy controls in the validation set were selected from a similar pool of controls recruited from April 2008 to July 2012.

The children included in the validation set were part of the Taiwan Birth Panel Study (Hsieh *et al*., 2011). At baseline, 486 mother–infant paired were enrolled from April 2004 to January 2005. Starting from the follow-up at 9 years, about 3 mL of venous blood was drawn and stored in RiboPure blood kit (Applied Biosystems/Life Technologies, Carlsbad, CA, USA) that was used for RNA extraction. A total of 66 children who completed the 9-year follow-up were included in the validation set.

These studies were approved by the Ethics Committees of the participating hospitals. Written informed consent was obtained from the father or the mother of the preterm infants and the adult participants themselves after a complete description of the study.

### RNA extraction and miRNA quantification

The blood specimens of all subjects were collected and processed within 3 h after blood drawing. For the adults, approximately 10 mL of whole blood was collected from each subject. Mononuclear leukocytes were separated by centrifuging with Ficoll-Paque PLUS (GE Healthcare). Then, the total RNA of these mononuclear leukocytes was extracted using TRIzol reagent (Invitrogen). For the preterm infants, the miRNAs were extracted from whole blood because of the small volume of blood collected (less than 500 μL). Arterial blood was collected from the participating infants prior to 36 weeks’ postmenstrual age in conjunction with a routine daily blood test. Approximately 2–4 μg of RNA from mononuclear leukocytes was extracted from each blood sample using the RiboPure™-Blood Kit (Applied Biosystems). The quality of the RNA extracts of both preterm infants and adults was good, that is, RNA integrity number > 8.

The expression of 365 human miRNAs was assayed using the Multiplex RT and TaqMan® Low Density Array (TLDA) Human MicroRNA Panel v1.0 and 7900 real-time RT–PCR System (Applied Biosystems), using RNU48 as an endogenous control. A total of 800 ng RNA was used for each array. Quantitative RT-PCR method with RNU48 as an endogenous control was used for the validation of individual miRNAs. The expression level of each miRNA from the array-based profiling was quantified using the normalized threshold cycle number (Δ*C*_t_), in which Δ*C*_t_ = [*C*_t_ (miRNA)] − [*C*_t_ (U48)], and the relative expression level was calculated as 2^−(ΔCt)^, which is commonly used in genome-wide miRNA profiling studies (Schmittgen *et al*., 2008).

### Statistical analyses

#### Differentially expressed miRNAs

Among the array-based expression levels of 365 miRNAs, only the 228 miRNAs that were detectable in ≥ 30% of the samples of either the preterm infants or adults were included for subsequent analyses. To select the differentially expressed miRNAs between preterm infants and adults, a two-sided Wilcoxon rank-sum test with Bonferroni correction was used to compare the continuous expression levels with a threshold *P*-value of 0.05/228 = 0.00022. On the basis of the previous research finding that human biological function and physical performance reach their peak at 20–35 years of age and wane afterward (Shephard, 1998), we chose 35 years of age as the cutoff point for middle-aged adulthood. When all of the adults were divided into young (≤ 35 years old, *n* = 32) and middle-aged adults (> 35 years old, *n* = 28), the expression levels between the groups were compared using a two-sided Wilcoxon rank-sum test with BH false discovery correction at the 20% level (Benjamini *et al*., 2001). The correlation/partial correlation analyses with Bonferroni correction (0.05/6 = 0.0083) were used to examine the correlation between differentially expressed miRNA and age in adults group. To help visualize the differential expression patterns, we adopted the method of matrix visualization using the software program Generalized Association Plots (Wu *et al*., 2010) to compare the miRNA expression levels between preterm infants and adults. The corresponding chromosomal locations of the miRNAs were retrieved from miRBase, release 19 (August 2012). For the validation set, ANOVAs with Bonferroni correction were used for three-group comparisons.

#### Functional network, microRNA Target Filter, and canonical pathway analysis using IPA

For the different classes of miRNAs and the miRANs at chromosomal hot spots, their functions were analyzed using ipa version 9.0 (release in June, 2012, Ingenuity® Systems, Redwood City, CA, USA). The purpose of the network algorithm was to show as many interactions as possible between the miRNAs of interest and their interactions at the molecular level. We used microRNA Target Filter and then performed canonical pathway analysis to identify the known-pathways involved by the miRNAs that were clustered on chromosomal hot spots.

In the network generating step, the miRNAs of interest, which interact with other molecules in the Ingenuity Knowledge Base, are considered Network Eligible miRNAs and serve as ‘seeds’ for generating networks. In the network, each connection represents known relationships between the molecules found in the Ingenuity Knowledge Base. The Network Score is based on the hypergeometric distribution and is calculated using the right-tailed Fisher’s exact test. The score takes into account the number of Network Eligible miRNAs in the network and its size (i.e. 35 molecules in our case), as well as the total number of Network Eligible miRNAs analyzed and the total number of molecules in the Ingenuity Knowledge Base that could potentially be included in the networks. For example, suppose that a network of 35 molecules has a Fisher’s exact test result of 1 × 10^−24^, and the network’s score is −log (Fisher’s exact test result) = 24. This can be interpreted as ‘there is a 1 in a million chance of obtaining a network containing at least the same number of Network Eligible miRNAs by chance when randomly picking 35 molecules from the Ingenuity Knowledge Base that could be in the networks’. Highly interconnected networks are likely to have significant biological functions and may be biologically significant in the experimental system. The right-tailed Fisher’s exact test was used to test the association between a biological function and/or a disease assigned to that network.

The microRNA Target Filter enables researchers for easy prioritization of experimentally validated and predicted mRNA targets from TargetScan, TarBase, miRecords, and the Ingenuity® Knowledge Base. We selected target genes that were experimentally observed or with high predicted confidence for the subsequent analyses. In canonical pathway analyses, the significance of the association between the dataset and the canonical pathway was determined based on two parameters: (a) a ratio of the number of genes from the dataset that map to the pathway vs. the total number of genes that map to the canonical pathway, and (b) a *P*-value calculated using Fisher’s exact test to determine the probability that the association between the genes in the dataset and the canonical pathway is due to chance alone.

## Supporting Information

**Fig. S1** The networks with the highest network’s score of different types of miRNAs: (A) nondifferentially expressed; (B) expressed in both groups, but down-regulated in adults; (C) expressed in both groups, but up-regulated in adults; and (D) decreased expression from young to middle-aged adulthood. The figure was drawn using IPA software. **Fig. S2** The scatter plot of miRNA expression levels on age in the adults (*n* = 60) of the screening set, with a fitted linear regression line displayed in solid line. **Fig. S3** The scatter plot of miRNA expression levels on age in the adults (*n* = 68) of the validation set, with a fitted linear regression line displayed in solid line. **Table S1** Demographic characteristics of the participants in the screening set and the validation set, respectively. **Table S2** The class of miRNAs with age-constant expression: the microRNAs (*n* = 104) that were nondifferentially expressed between preterm-infants (*n* = 30) and adults (*n* = 60), and their chromosomal locations. **Table S3** The class of miRNAs with age-limited expression: the miRNAs (*n* = 23) that were expressed either in preterm-infants only or in adults only, and their chromosomal locations. **Table S4** The class of miRNAs with age-related modulation-down regulated in adults: the miRNAs (*n* = 20) that were differentially expressed between preterm-infants (*n* = 30) and adults (*n* = 60), down-regulated in adults, and their chromosomal locations. **Table S5** The class of miRNAs with age-related modulation-up regulated in adults: the miRNAs (*n* = 81) that were differentially expressed between preterm-infants (*n* = 30) and adults (*n* = 60), up-regulated in adults, and their chromosomal locations. **Table S6** Distribution of chromosomal locations for detectable miRNAs. **Table S7** The detailed chromosomal loci of miRNAs located in four chromosomes that showed a higher proportion for a certain class of miRNAs than the averaged ones as shown in Table S6. **Table S8** Top five associated canonical pathways for predicted miR-target genes of the miRNA that were clustered on 14q32.31 and 9q22. **Table S9** The target genes derived from the top five associated canonical pathways for the age-constant expression miRNAs clustered on 14q32.31. **Table S10** The target genes derived from the top five associated canonical pathways for the age-related up-regulation expression in adults miRNAs clustered on 9q22.32.
