# Supplementary material for: Modulated expression of human peripheral blood microRNAs from infancy to adulthood and its role in aging
Source: Aging Cell. 2014 May 6;13(4):679–89. doi: 10.1111/acel.12225 (PMC4326935; doi:10.1111/acel.12225)
Supplement: Supplementary file 1 — Fig. S1 The networks with the highest network’s score of different types of miRNAs: (A) nondifferentially expressed; (B) expressed in both groups, but down-regulated in adults; (C) expressed in both groups, but up-regulated in adults; and (D) decreased expression from young to middle-aged adulthood. The figure was drawn using IPA software. Fig. S2 The scatter plot of miRNA expression levels on age in the adults (n = 60) of the screening set, with a fitted linear regression line displayed in solid line. Fig. S3 The scatter plot of miRNA expression levels on age in the adults (n = 68) of the validation set, with a fitted linear regression line displayed in solid line. Table S1 Demographic characteristics of the participants in the screening set and the validation set, respectively. Table S2 The class of miRNAs with age-constant expression: the microRNAs (n = 104) that were nondifferentially expressed between preterm-infants (n = 30) and adults (n = 60), and their chromosomal locations. Table S3 The class of miRNAs with age-limited expression: the miRNAs (n = 23) that were expressed either in preterm-infants only or in adults only, and their chromosomal locations. Table S4 The class of miRNAs with age-related modulation-down regulated in adults: the miRNAs (n = 20) that were differentially expressed between preterm-infants (n = 30) and adults (n = 60), down-regulated in adults, and their chromosomal locations. Table S5 The class of miRNAs with age-related modulation-up regulated in adults: the miRNAs (n = 81) that were differentially expressed between preterm-infants (n = 30) and adults (n = 60), up-regulated in adults, and their chromosomal locations. Table S6 Distribution of chromosomal locations for detectable miRNAs. Table S7 The detailed chromosomal loci of miRNAs located in four chromosomes that showed a higher proportion for a certain class of miRNAs than the averaged ones as shown in Table S6. Table S8 Top five associated canonical pathways for predicted miR-ta [file acel0013-0679-sd1.doc]

**Supporting Information**

| **Contents** | page |
| --- | --- |
| **Fig. S1** The networks with the highest network’s score of different types of miRNAs: (A) non-differentially expressed; (B) expressed in both groups, but down-regulated in adults; (C) expressed in both groups, but up-regulated in adults; and (D) decreased expression from young to middle-aged adulthood. The figure was drawn using IPA software. | 3 |
|  |  |
| **Fig. S2** The scatter plot of miRNA expression levels on age in the adults (n = 60) of the screening set, with a fitted linear regression line displayed in solid line. The expression levels were presented using -∆Ct. In the legend, r denotes Pearson correlation coefficient, and adjusted r denotes Partial correlation coefficient with adjustment for gender | 6 |
|  |  |
| **Fig. S3** The scatter plot of miRNA expression levels on age in the adults (n = 68) of the validation set, with a fitted linear regression line displayed in solid line. The expression levels were presented using -∆Ct. In the legend, r denotes Pearson correlation coefficient, and adjusted r denotes Partial correlation coefficient with adjustment for gender. | 7 |
|  |  |
| **Table S1**. Demographic characteristics of the participants in the screening set and the validation set, respectively. | 8 |
|  |  |
| **Table S2.** The class of miRNAs with age-constant expression: the microRNAs (n = 104) that were non-differentially expressed between preterm-infants (n = 30) and adults (n = 60), and their chromosomal locations. | 9 |
|  |  |
| **Table S3.** The class of miRNAs with age-limited expression: the miRNAs (n = 23) that were expressed either in preterm-infants only or in adults only, and their chromosomal locations. | 13 |
|  |  |
| **Table S4.** The class of miRNAs with age-related modulation−down regulated in adults: the miRNAs (n = 20) that were differentially expressed between preterm-infants (n = 30) and adults (n = 60), down-regulated in adults, and their chromosomal locations. | 14 |
|  |  |
| **Table S5.** The class of miRNAs with age-related modulation−up regulated in adults: the miRNAs (n = 81) that were differentially expressed between preterm-infants (n = 30) and adults (n = 60), up-regulated in adults, and their chromosomal locations. | 15 |
|  |  |
| **Table S6**. Distribution of chromosomal locations for detectable miRNAs. | 18 |
|  |  |
| **Table S7.** The detailed chromosomal loci of miRNAs located in four chromosomes that showed a higher proportion for a certain class of miRNAs than the averaged ones as shown in Table S6. | 19 |
|  |  |
| **Table S8**. Top five associated canonical pathways for predicted miR-target genes of the miRNA that were clustered on14q32.31 and 9q22. | 20 |
|  |  |
| **Table S9**. The target genes derived from the top five associated canonical pathways for the age-constant expression miRNAs clustered on 14q32.31. | 21 |
|  |  |
| **Table S10.** The target genes derived from the top five associated canonical pathways for the the age-related up-regulation expression in adults miRNAs clustered on 9q22.32. | 32 |

**Fig. S1** The networks with the highest network’s score of different types of miRNAs: (A) non-differentially expressed; (B) expressed in both groups, but down-regulated in adults; (C) expressed in both groups, but up-regulated in adults; and (D) decreased expression from young to middle-aged adulthood. The figure was drawn using IPA software.

**(A)**


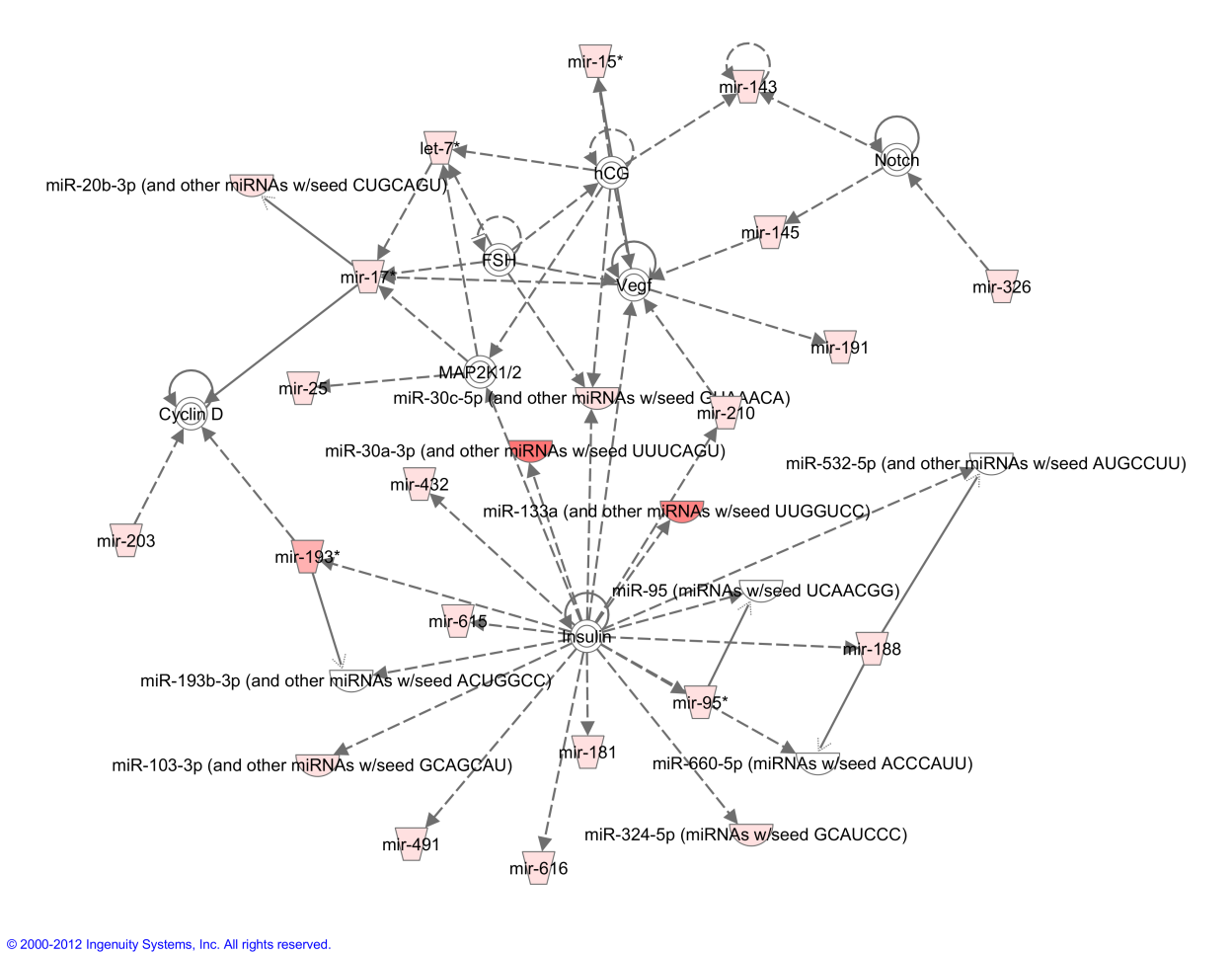


**(**B**)**


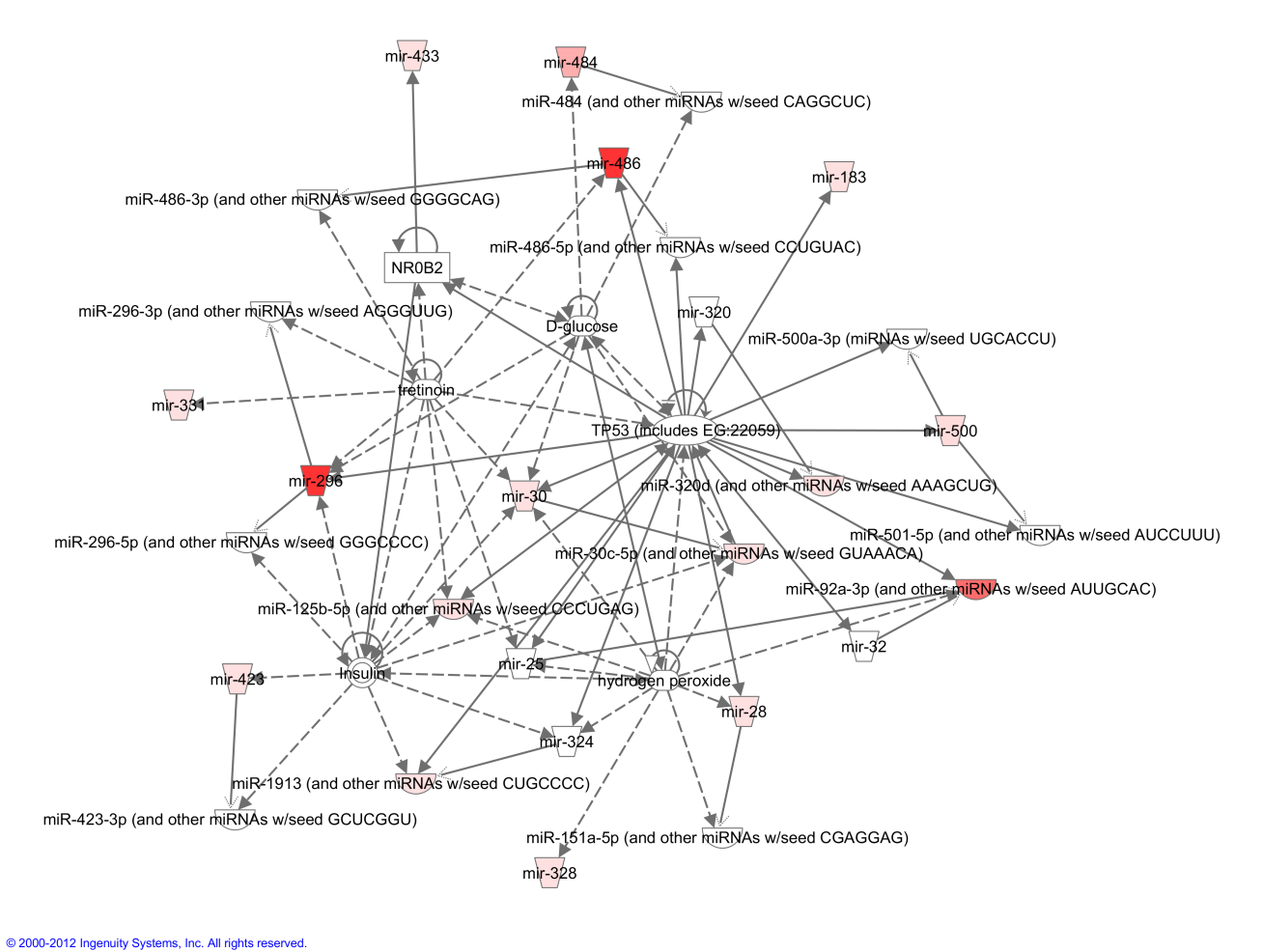


C)


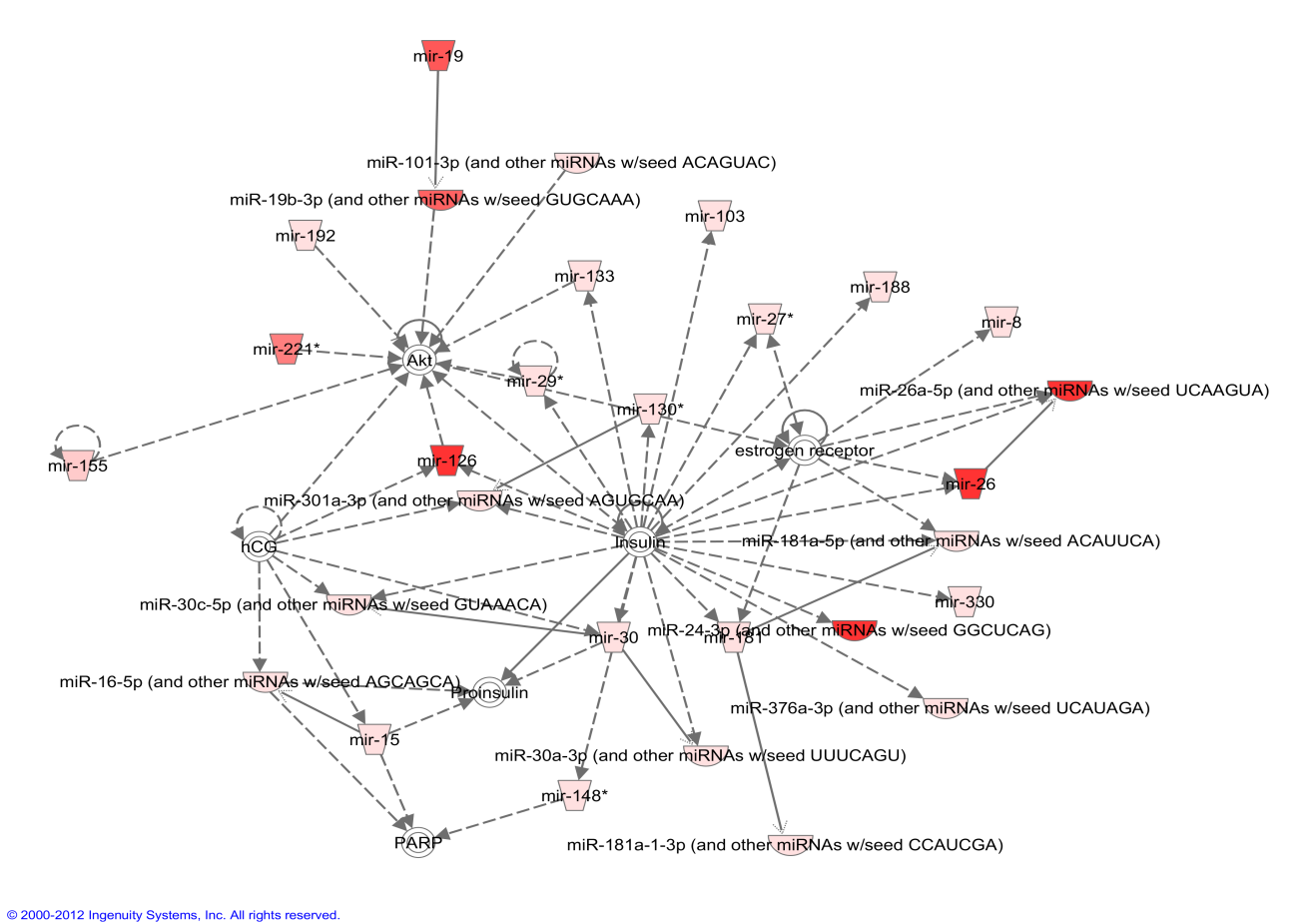


**(D)**


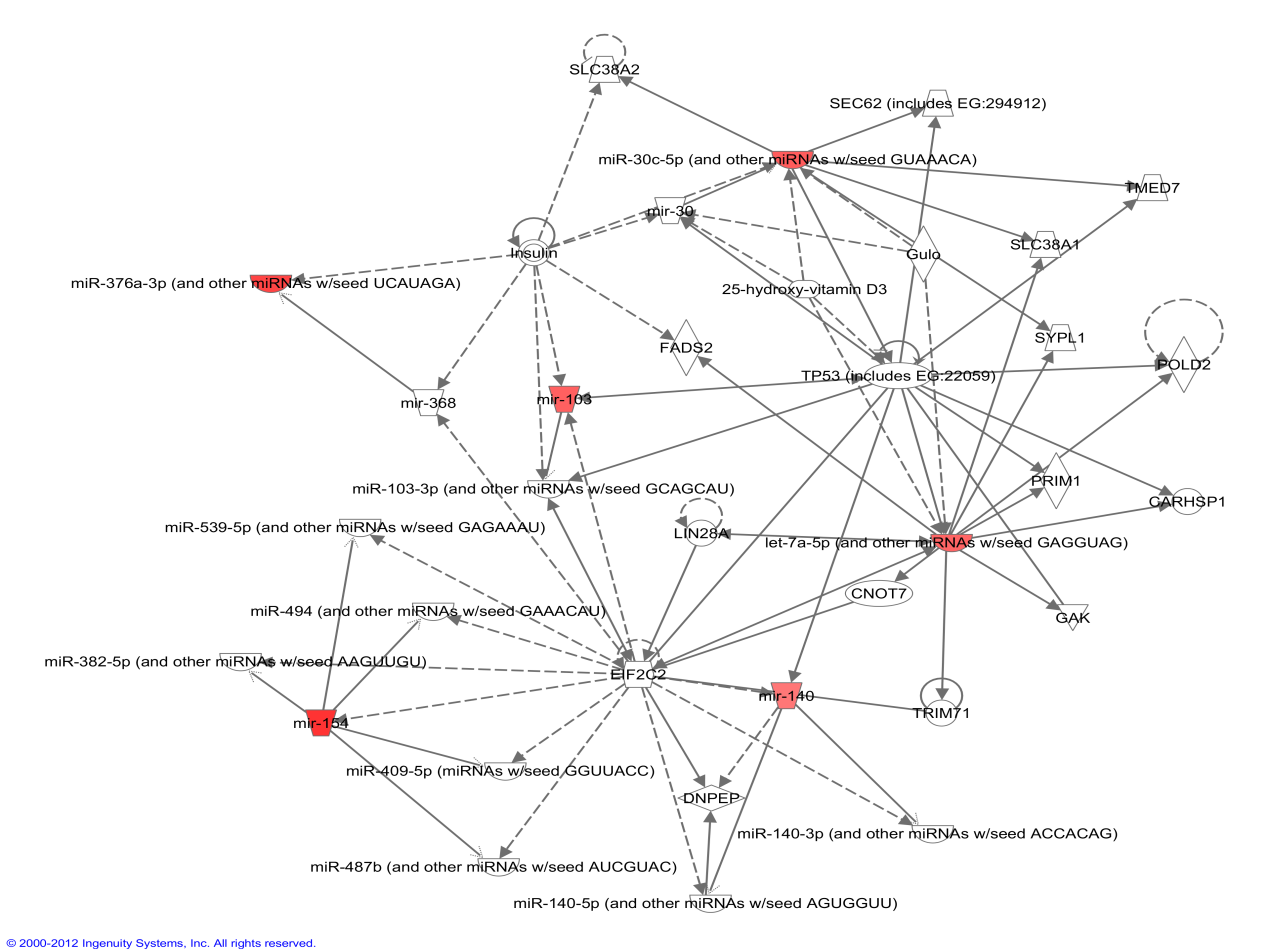


**Fig. S2** The scatter plot of miRNA expression levels on age in the adults (n = 60) of the screening set, with a fitted linear regression line displayed in solid line. The expression levels were presented using -∆Ct. In the legend, r denotes Pearson correlation coefficient, and adjusted r denotes Partial correlation coefficient with adjustment for gender.

**
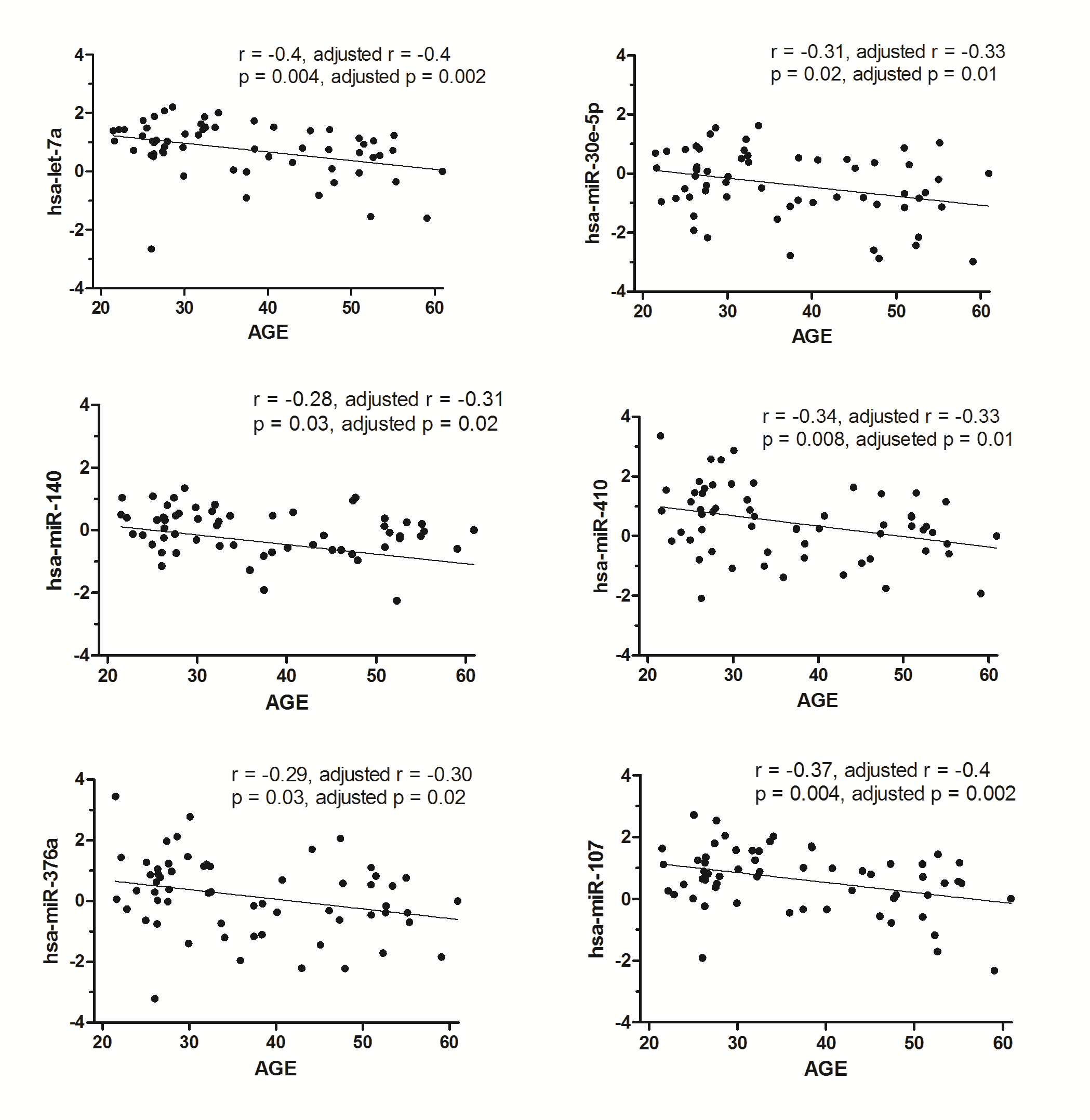
**

**Fig. S3** The scatter plot of miRNA expression levels on age in the adults (n = 68) of the validation set, with a fitted linear regression line displayed in solid line. The expression levels were presented using -∆Ct. In the legend, r denotes Pearson correlation coefficient, and adjusted r denotes Partial correlation coefficient with adjustment for gender.

**
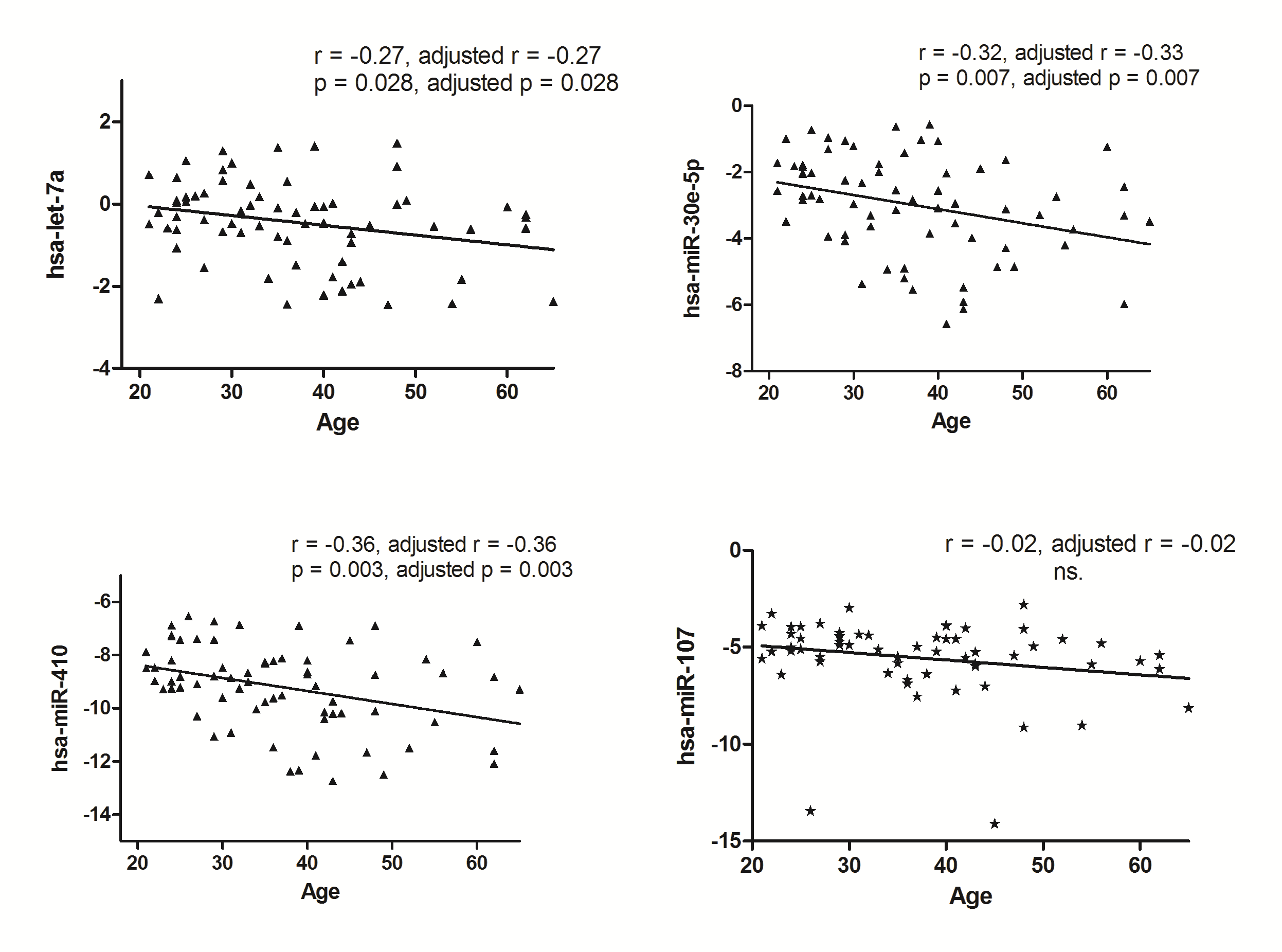
**

Table S1. Demographic characteristics of the participants in the screening set and the validation set, respectively.

| Variables | Screening set | | |  | Validation set | | | |
| --- | --- | --- | --- | --- | --- | --- | --- | --- |
|  | n (%) | | |  | n (%) | | | |
| Preterm infants | (N = 30) | | |  | (N = 22) | | | |
| Age (weeks) | Mean = 29.1, SD = 2.4 | | |  | Mean = 28.6, SD = 2.4 | | | |
| 24-35 | 30 | (100) | |  | 22 | (100) | | |
| Gender |  | |  |  |  |  | | |
| Male | 17 | (56.7) | |  | 12 | (55.0) | | |
| Female | 13 | (43.3) | |  | 10 | (45.0) | | |
| Children | (not applicable) | | |  | (N = 66) | | | |
| Age (years) | - | | |  | Mean = 9.1, SD = 0.1 | | | |
| 9-10 | - | | |  | 66 | | (100) | |
| Gender | - | | |  |  | | |  |
| Male | - | | |  | 31 | | (47.0) | |
| Female | - | | |  | 35 | | (53.0) | |
| Adults | (N = 60) | | |  | (N = 68) | | | |
| Age (years) | Mean = 37, SD = 11.7 | | |  | Mean = 36.9, SD = 11.4 | | | |
| 21-25 | 7 | (11.7) | |  | 14 | | (20.6) | |
| 26-30 | 17 | (28.3) | |  | 10 | | (14.7) | |
| 31-35 | 8 | (13.3) | |  | 10 | | (14.7) | |
| 36-40 | 5 | ( 8.3) | |  | 11 | | (16.2) | |
| 41-45 | 4 | ( 6.7) | |  | 9 | | (13.2) | |
| 46-50 | 6 | (10.0) | |  | 5 | | ( 7.4) | |
| 51-55 | 9 | (15.0) | |  | 3 | | ( 4.4) | |
| 56-60 | 1 | ( 5.0) | |  | 2 | | ( 2.9) | |
| 61-65 | 1 | ( 1.7) | |  | 4 | | ( 5.9) | |
| Gender |  |  | |  |  | |  | |
| Male | 24 | (40.0) | |  | 23 | | (33.8) | |
| Female | 26 | (60.0) | |  | 45 | | (66.0) | |

**Table S2.** The class of miRNAs with age-constant expression: the microRNAs (n = 104) that were non-differentially expressed between preterm-infants (n = 30) and adults (n = 60), and their chromosomal locations.

| miRNA name^a^ | Chromosomal Location | Chromosomal coordinates ^b^ |
| --- | --- | --- |
| hsa-miR-197 | 1p13.3 | 1: 110141515-110141589 [+] |
| hsa-miR-200a | 1p36.33 | 1: 1103243-1103332 [+] |
| hsa-miR-429 | 1p36.33 | 1: 1104385-1104467 [+] |
| hsa-miR-215 | 1q41 | 1: 220291195-220291304 [+] |
| hsa-miR-194 | 1q41 | 1: 220291499-220291583 [+] |
|  | 11q13.1 | 11: 64658827-64658911 [+] |
| hsa-miR-200b | 1p36.33 | 1: 1102484-1102578 [+] |
| hsa-miR-214 | 1q24.3 | 1: 172107938-172108047 [-] |
| hsa-miR-34a | 1p36.22|1p36.22 | 1: 9211727-9211836 [-] |
| hsa-miR-137 | 1p21.3 | 1: 98511626-98511727 [-] |
| hsa-miR-149 | 2q37.3 | 2: 241395418-241395506 [+] |
| hsa-miR-15b | 3q25.33 | 3: 160122376-160122473 [+] |
| hsa-miR-564 | 3p21.31 | 3: 44903380-44903473 [+] |
| hsa-miR-425-5p | 3p21.31 | 3: 49057581-49057667 [+] |
| hsa-miR-135a | 3p21.1 | 3: 52328235-52328324 [+] |
|  | 12q23.1 | 12: 97957590-97957689 [+] |
| hsa-miR-425 | 3p21.31 | 3: 49057581-49057667 [-] |
| hsa-miR-191 | 3p21.31 | 3: 49058051-49058142 [-] |
| hsa-miR-572 | 4p15.33 | 4: 11370451-11370545 [+] |
| hsa-miR-218 | 4p15.31 | 4: 20529898-20530007 [+] |
|  | 5q34 | 5: 168195151-168195260 [+] |
| hsa-miR-95 | 4 | 4: 8007028-8007108 [+] |
| hsa-miR-378a | 5q32 | 5: 149112388-149112453 [+] |
| hsa-miR-422b | 5q32 | 5: 149112388-149112453 [+] |
| hsa-miR-449a | 5q11.2 | 5: 54466360-54466450 [+] |
| hsa-miR-449b | 5q11.2 | 5: 54466474-54466570 [+] |
| hsa-miR-143 | 5q32 | 5: 148808481-148808586 [+] |
| hsa-miR-145 | 5q32 | 5: 148810209-148810296 [+] |
| hsa-miR-579 | 5p13.3 | 5: 32394484-32394581 [-] |
| hsa-miR-30c | 6q13 | 6: 72086663-72086734 [+] |
|  | 1p34.2 | 1: 41222956-41223044 [+] |
| hsa-miR-30a-3p | 6q13 | 6: 72113254-72113324 [+] |
| hsa-miR-182 | 7q32.2 | 7: 129410223-129410332 [-] |
| hsa-miR-589 | 7p22.1 | 7: 5535450-5535548 [+] |
| hsa-miR-25 | 7q22.1 | 7: 99691183-99691266 [+] |
| hsa-miR-93 | 7q22.1 | 7: 99691391-99691470 [-] |
| hsa-miR-548d | 8q24.13 | 8: 124360274-124360370 [+] |
|  | 17q24.2 | 17: 65467605-65467701 [+] |
| hsa-miR-597 | 8p23.1 | 8: 9599182-9599278 [+] |
| hsa-miR-199b | 9q34.11 | 9: 131007000-131007109 [+] |
| hsa-miR-491 | 9p21.3 | 9: 20716104-20716187 [+] |
| hsa-miR-204 | 9q21.12 | 9: 73424891-73425000 [+] |
| hsa-miR-202 | 10q26.3 | 10: 135061015-135061124 [-] |
| hsa-miR-511 | 10p12.33 | 10: 17887107-17887193 [+] |
|  | 10p12.33 | 10: 18134036-18134122 [+] |
| hsa-miR-34b | 11q23.1 | 11: 111383663-111383746 [+] |
| hsa-miR-100 | 11q24.1 | 11: 122022937-122023016 [+] |
| hsa-miR-210 | 11p15.5 | 11: 568089-568198 [+] |
| hsa-miR-326 | 11q13.4 | 11: 75046136-75046230 [+] |
| hsa-miR-139 | 11q13.4 | 11: 72326107-72326174 [-] |
| hsa-miR-616 | 12q13.3 | 12: 57912946-57913042 [+] |
| hsa-miR-141 | 12p13.31 | 12: 7073260-7073354 [+] |
| hsa-miR-618 | 12q21.31 | 12: 81329515-81329612 [+] |
| hsa-miR-615 | 12q13.13 | 12: 54427734-54427829 [+] |
| hsa-miR-15a | 13q14.2 | 13: 50623255-50623337 [+] |
| hsa-miR-17-3p | 13q31.3 | 13: 92002859-92002942 [+] |
| hsa-miR-18a | 13q31.3 | 13: 92003005-92003075 [+] |
| hsa-miR-493 | 14q32.2 | 14: 101335397-101335485 [+] |
| hsa-miR-127 | 14q32.2 | 14: 101349316-101349412 [+] |
| hsa-miR-432 | 14q32.2 | 14: 101350820-101350913 [+] |
| hsa-miR-299-5p | 14q32.31 | 14: 101490131-101490193 [+] |
| hsa-miR-329 | 14q32.31 | 14: 101493122-101493201 [+] |
|  | 14q32.31 | 14: 101493437-101493520 [+] |
| hsa-miR-654 | 14q32.31 | 14: 101506556-101506636 [+] |
| hsa-miR-381 | 14q32.31 | 14: 101512257-101512331 [+] |
| hsa-miR-485-5p | 14q32.31 | 14: 101521756-101521828 [+] |
| hsa-miR-485-3p | 14q32.31 | 14: 101521756-101521828 [+] |
| hsa-miR-412 | 14q32.31 | 14: 101531784-101531874 [+] |
| hsa-miR-369-5p | 14q32.31 | 14: 101531935-101532004 [+] |
| **hsa-miR-410** | 14q32.31 | 14: 101532249-101532328 [+] |
| hsa-miR-656 | 14q32.31 | 14: 101533061-101533138 [+] |
| hsa-miR-203 | 14q32.33 | 14: 104583742-104583851 [+] |
| hsa-miR-624 | 14q12 | 14: 31483852-31483948 [+] |
| hsa-miR-345 | 14q32.2 | 14: 100774196-100774293 [+] |
| hsa-miR-379 | 14q32.31 | 14: 101488403-101488469 [+] |
| hsa-miR-323 | 14q32.31 | 14: 101492069-101492154 [+] |
| hsa-miR-494 | 14q32.31 | 14: 101495971-101496051 [+] |
| hsa-miR-376a* | 14q32.31 | 14: 101507119-101507186 [+] |
| hsa-miR-496 | 14q32.31 | 14: 101526910-101527011 [+] |
| hsa-miR-409-5p | 14q32.31 | 14: 101531637-101531715 [+] |
| hsa-miR-211 | 15q13.3 | 15: 31357235-31357344 [+] |
| hsa-miR-627 | 15q15.1 | 15: 42491768-42491864 [+] |
| hsa-miR-190 | 15q22.2 | 15: 63116156-63116240 [+] |
| hsa-miR-629-3p | 15q23 | 15: 70371711-70371807 [+] |
| hsa-miR-422a | 15q22.31 | 15: 64163129-64163218 [-] |
| hsa-miR-193b | 16p13.12 | 16: 14397824-14397906 [+] |
| hsa-miR-10a | 17q21.32 | 17: 46657200-46657309 [+] |
| hsa-miR-497 | 17p13.1 | 17: 6921230-6921341 [+] |
| hsa-miR-324-5p | 17p13.1 | 17: 7126616-7126698 [+] |
| hsa-miR-451 | 17q11.2 | 17: 27188387-27188458 [-] |
| hsa-miR-193a | 17q11.2 | 17: 29887015-29887102 [+] |
| hsa-miR-133a | 18q11.2 | 18: 19405659-19405746 [-] |
|  | 20q13.33 | 20: 61162119-61162220 [+] |
| hsa-miR-187 | 18q12.2 | 18: 33484781-33484889 [-] |
| hsa-miR-199a | 19p13.2 | 19: 10928102-10928172 [+] |
|  | 1q24.3 | 1: 172113675-172113784 [+] |
| hsa-miR-181c | 19p13.13 | 19: 13985513-13985622 [+] |
| hsa-let-7e | 19q13.41 | 19: 52196039-52196117 [+] |
| hsa-miR-103 | 5q34 | 5: 167987901-167987978 [+] |
|  | 20p13 | 20: 3898141-3898218 [+] |
| hsa-miR-99a | 21q21.1 | 21: 17911409-17911489 [+] |
| hsa-let-7c | 21q21.1 | 21: 17912148-17912231 [+] |
| hsa-miR-650 | 22q11.22 | 22: 23165270-23165365 [+] |
| hsa-miR-363* | Xq26.2 | X: 133303408-133303482 [+] |
| hsa-miR-20b | Xq26.2 | X: 133303839-133303907 [+] |
| hsa-miR-503 | Xq26.3 | X: 133680358-133680428 [+] |
| hsa-miR-452 | Xq28 | X: 151128100-151128184 [+] |
| hsa-miR-532 | Xp11.23 | X: 49767754-49767844 [+] |
| hsa-miR-362 | Xp11.23 | X: 49773572-49773636 [+] |
| hsa-miR-502 | Xp11.23 | X: 49779206-49779291 [+] |
| hsa-miR-542-5p | Xq26.3 | X: 133675371-133675467 [-] |
| hsa-miR-452 | Xq28 | X: 151128100-151128184 [+] |
| hsa-miR-545 | Xq13.2 | X: 73506939-73507044 [+] |
| hsa-miR-594 | N/A | N/A |

^a^ The bold miRNA was associated with aging.

^b^ [+]: forward strands; [-]: reverse strand

**Table S3**. The class of miRNAs with age-limited expression: the miRNAs (n = 23) that were expressed either in preterm-infants only or in adults only, and their chromosomal locations.

| miRNA name | Expression group | Chromosomal Location | Chromosomal coordinates ^a^ |
| --- | --- | --- | --- |
| hsa-miR-325 | Infants | Xq21.1 | X: 76225829-76225926 [+] |
| hsa-miR-9* | Adults | 1q22 | 1: 156390133-156390221 [+] |
|  |  | 5q14.3 | 5: 87962671-87962757 [+] |
|  |  | 15q26.1 | 15: 89911248-89911337 [+] |
| hsa-miR-556-5p | Adults | 1q23.3 | 1: 162312336-162312430 [+] |
| hsa-miR-135b | Adults | 1q32.1 | 1: 205417430-205417526 [+] |
| hsa-miR-10b | Adults | 2q31.1 | 2: 177015031-177015140 [+] |
| hsa-miR-153 | Adults | 2q35 | 2: 220158833-220158922 [+] |
|  |  | 7q36.3 | 7: 157367028-157367114 [+] |
| hsa-miR-551b | Adults | 3q26.2 | 3: 168269642-168269737 [+] |
| hsa-miR-580 | Adults | 5p13.2 | 5: 36147994-36148090 [+] |
| hsa-miR-548a | Adults | 6p22.3 | 6: 18572015-18572111 [+] |
|  |  | 6q23.3 | 6: 135560298-135560394 [+] |
|  |  | 8q22.3 | 8: 105496597-105496693 [+] |
| hsa-miR-219 | Adults | 6p21.32 | 6: 33175612-33175721 [+] |
|  |  | 9q34.11 | 9: 131154897-131154993 [+] |
| hsa-miR-196a | Adults | 17q21.32 | 12: 54385522-54385631 [+] |
|  |  | 12q13.13 | 17: 46709852-46709921 [+] |
| hsa-miR-380-5p | Adults | 14q32.31 | 14: 101491354-101491414 [+] |
| hsa-miR-369-3p | Adults | 14q32.31 | 14: 101531935-101532004 [+] |
| hsa-miR-338 | Adults | 17q25.3 | 17: 79099683-79099749 [+] |
| hsa-miR-1 | Adults | 20q13.33 | 18: 19408965-19409049 [+] |
|  |  | 18q11.2 | 20: 61151513-61151583 [+] |
| hsa-miR-189 | Adults | NA | NA |
| hsa-miR-651 | Adults | Xp22.31 | X: 8095006-8095102 [+] |
| hsa-miR-652-3p^b^ | Adults | Xq23 | X: 109298557-109298654 [+] |
| hsa-miR-450a | Adults | Xq26.3 | X: 133674371-133674461 [+] |
|  |  | Xq26.3 | X: 133674538-133674637 [+] |
| hsa-miR-424 | Adults | Xq26.3 | X: 133680644-133680741 [+] |
| hsa-miR-509 | Adults | Xq27.3 | X: 146342050-146342143 [-] |
| hsa-miR-514a | Adults | Xq27.3 | [X: 146360765-146360862 [-]](http://www.ensembl.org/Homo_sapiens/contigview?l=X:146358765-146362862) |
|  |  | Xq27.3 | [X: 146363461-146363548 [-]](http://www.ensembl.org/Homo_sapiens/contigview?l=chrX:146361461-146365548) |
|  |  | Xq27.3 | [X: 146366159-146366246 [-]](http://www.ensembl.org/Homo_sapiens/contigview?l=chrX:146364159-146368246) |

^a^ [+]: forward strands; [-]: reverse strand

NA = not available in database miRBase, release 19 in August 2012.

^b^ Reported to be up-regulated in schizophrenia.

**Table S4.** The class of miRNAs with age-related modulation−down regulated in adults: the miRNAs (n = 20) that were differentially expressed between preterm-infants (n = 30) and adults (n = 60), down-regulated in adults, and their chromosomal locations.

| miRNA name^a^ | Up- or Down- regulated among adults | P**^b^** value | Chromosomal Location | | Chromosomal coordinatesc |
| --- | --- | --- | --- | --- | --- |
| hsa-miR-125b | Down | 8.1E-05 | 11q24.1 | 11: 121970465-121970552 [-] | |
|  |  |  | 21q21.1 | 21: 17962557-17962645 [+] | |
| hsa-miR-331 | Down | 1.6E-08 | 12q22 | 12: 95702196-95702289 [+] | |
| hsa-miR-92a | Down | 1.9E-14 | 13q31.3 | 13: 92003568-92003645 [+] | |
|  |  |  | Xq26.2 | X: 133303568-133303642 [-] | |
| hsa-miR-432* | Down | 8.2E-06 | 14q32.2 | 14: 101350820-101350913 [+] | |
| hsa-miR-433 | Down | 7.3E-09 | 14q32.2 | 14: 101348223-101348315 [+] | |
| hsa-miR-484 | Down | 3.5E-14 | 16p13.11 | 16: 15737151-15737229 [+] | |
| hsa-miR-328 | Down | 1.1E-08 | 16q22.1 | 16: 67236224-67236298 [-] | |
| hsa-miR-423 | Down | 1.2E-10 | 17q11.2 | 17: 28444097-28444190 [+] | |
| hsa-miR-324-3p | Down | 1.2E-13 | 17p13.1 | 17: 7126616-7126698 [-] | |
| hsa-miR-296 | Down | 1.4E-14 | 20q13.32 | 20: 57392670-57392749 [-] | |
| hsa-miR-185 | Down | 7.9E-06 | 22q11.21 | 22: 20020662-20020743 [+] | |
| hsa-miR-28-5p^d^ | Down | 1.4E-11 | 3q28 | 3: 188406569-188406654 [+] | |
| hsa-miR-30a-5p | Down | 0.0002 | 6q13 | 6: 72113254-72113324 [+] | |
| hsa-miR-183 | Down | 2.4E-09 | 7q32.2 | 7: 129414745-129414854 [-] | |
| hsa-miR-550a-3p | Down | 1.4E-09 | 7p14.3 | 7: 30329410-30329506 [+] | |
|  |  |  | 7p14.3 | 7: 32772593-32772689 [+] | |
|  |  |  | 7 | 7: 29720350-29720444 [-] | |
| hsa-miR-320a | Down | 4.3E-11 | 8p21.3 | 8: 22102475-22102556 [+] | |
| hsa-miR-30d | Down | 1E-06 | 8q24.22 | 8: 135817119-135817188 [-] | |
| hsa-miR-486-5p | Down | 1.4E-14 | 8p11.21 | 8: 41517959-41518026 [-] | |
| hsa-miR-500a | Down | 1.6E-05 | Xp11.23 | X: 49773039-49773122 [+] | |
| hsa-miR-501 | Down | 8.1E-14 | Xp11.23 | X: 49774330-49774413 [+] | |
|  |  |  | 9p24.1 | 9: 4850297-4850375 [+] | |

^a^ The bold miRNA was associated with aging.

^b^ p<0.00024 based on the Wilcoxon rank-sum test with Bonferroni correction

^c^ [+]: forward strands; [-]: reverse strand

^d^ Reported to be down-regulated in schizophrenia.

**Table S5**. The class of miRNAs with age-related modulation−up regulated in adults: the miRNAs (n = 81) that were differentially expressed between preterm-infants (n = 30) and adults (n = 60), up-regulated in adults, and their chromosomal locations.

| miRNA name^a^ | Up- or Down- regulated among adults | P**^b^** value | Chromosomal Location | | Chromosomal coordinates^c^ |
| --- | --- | --- | --- | --- | --- |
| hsa-miR-181a^d^ | Up | 3.1E-09 | 9q33.3 | 1: 198828173-198828282 [+] | |
| hsa-miR-30e-3p^d^ | Up | 9.9E-13 | 1p34.2 | 1: 41220027-41220118 [+] | |
| **hsa-miR-30e-5p** | Up | 8.2E-10 | 1p34.2 | 1: 41220027-41220118 [+] | |
| hsa-miR-9 | Up | 8.5E-05 | 1q22 | 1: 156390133-156390221 [-] | |
|  |  |  | 5q14.3 | 5: 87962671-87962757 [-] | |
| hsa-miR-181b | Up | 1.9E-13 | 1q32.1 | 1: 198828002-198828111 [-] | |
|  |  |  | 9q33.3 | 9: 127455989-127456077 [+] | |
| hsa-miR-29c | Up | 1E-12 | 1q32.2 | 1: 207975197-207975284 [-] | |
| hsa-miR-101 | Up | 8.6E-13 | 1p31.3 | 1: 65524117-65524191 [-] | |
|  |  |  | 9p24.1 | 9: 4850297-4850375 [+] | |
| hsa-miR-186 | Up | 3.3E-14 | 1p31.1 | 1: 71533314-71533399 [-] | |
| hsa-miR-26a | Up | 1.4E-14 | 3p22.2 | 3: 38010895-38010971 [+] | |
|  |  |  | 12q14.1 | 12: 58218392-58218475 [-] | |
| hsa-let-7g | Up | 2.2E-14 | 3p21.1 | 3: 52302294-52302377 [-] | |
| hsa-miR-576-5p^d^ | Up | 0.00024 | 4 | 4: 110409854-110409951 [+] | |
| hsa-miR-146a | Up | 5.4E-14 | 5q34 | 5: 159912359-159912457 [+] | |
| hsa-miR-340 | Up | 7.8E-12 | 5q35.3 | 5: 179442303-179442397 [-] | |
| hsa-miR-133b ^e^ | Up | 1.9E-07 | 6p12.2 | 6: 52013721-52013839 [+] | |
| hsa-miR-339 | Up | 8.2E-09 | 7p22.3 | 7: 1062569-1062662 [-] | |
| hsa-miR-335-5p | Up | 5E-07 | 7q32.2 | 7: 130135952-130136045 [+] | |
| hsa-miR-29a | Up | 4.9E-13 | 7q32.3 | 7: 130561506-130561569 [-] | |
| hsa-miR-148a | Up | 6.5E-13 | 7p15.2 | 7: 25989539-25989606 [-] | |
| hsa-miR-196b | Up | 2.5E-09 | 7p15.2 | 7: 27209099-27209182 [-] | |
| hsa-miR-106b | Up | 1.1E-11 | 7q22.1 | 7: 99691616-99691697 [-] | |
| hsa-miR-30b | Up | 2.6E-13 | 8q24.22 | 8: 135812763-135812850 [-] | |
| hsa-miR-151-3p^d^ | Up | 2.2E-14 | 8q24.3 | 8: 141742663-141742752 [-] | |
| hsa-miR-126* | Up | 1.2E-11 | 9q34.3 | 9: 139565054-139565138 [+] | |
| hsa-miR-32 | Up | 2.9E-10 | 9q31.3 | 9: 111808509-111808578 [-] | |
| hsa-miR-126 | Up | 1.4E-14 | 9q34.3 | 9: 139565054-139565138 [+] | |
| hsa-miR-31^d^ | Up | 1.6E-06 | 9p21.3 | 9: 21512114-21512184 [-] | |
| hsa-miR-7 ^e^ | Up | 1.1E-06 | 9q21.32 | 9: 86584663-86584772 [-] | |
|  |  |  | 15q26.1 | 15: 89155056-89155165 [+] | |
|  |  |  | 19p13.3 | 19: 4770682-4770791 [+] | |
| hsa-let-7f | Up | 6.2E-14 | 9q22.32 | 9: 96938629-96938715 [+] | |
|  |  |  | Xp11.22 | X: 53584153-53584235 [-] | |
| hsa-miR-24 | Up | 1.4E-14 | 9q22.32 | 9: 97848303-97848370 [+] | |
|  |  |  | 19p13.13 | 19: 13947101-13947173 [-] | |
| **hsa-let-7a** | Up | 1.1E-11 | 9q22.32 | 9: 96938239-96938318 [+] | |
|  |  |  | 11q24.1 | 11: 122017230-122017301 [-] | |
|  |  |  | 22q13.31 | 22: 46508629-46508702 [+] | |
| hsa-let-7d | Up | 3E-09 | 9q22.32 | 9: 96941116-96941202 [+] | |
| hsa-miR-23b | Up | 1.9E-07 | 9q22.32 | 9: 97847490-97847586 [+] | |
| hsa-miR-27b | Up | 1.8E-05 | 9q22.32 | 9: 97847727-97847823 [+] | |
| hsa-miR-146b | Up | 5.4E-14 | 10q24.32 | 10: 104196269-104196341 [+] | |
| **hsa-miR-107**^d^ | Up | 2E-08 | 10q23.31 | 10: 91352504-91352584 [-] | |
| hsa-miR-130a | Up | 1.3E-10 | 11q12.1 | 11: 57408671-57408759 [+] | |
| hsa-miR-192 | Up | 1.3E-07 | 11q13.1 | 11: 64658609-64658718 [-] | |
| hsa-miR-148b^d^ | Up | 3.1E-11 | 12q13.13 | 12: 54731000-54731098 [+] | |
| hsa-miR-200c^d^ | Up | 5.9E-10 | 12p13.31 | 12: 7072862-7072929 [+] | |
| hsa-miR-17-5p | Up | 1.7E-13 | 13q31.3 | 13: 92002859-92002942 [+] | |
| hsa-miR-16 | Up | 2.6E-07 | 13q14.2 | 13: 50623109-50623197 [-] | |
|  |  |  | 3q25.33 | 3: 160122533-160122613 [+] | |
| hsa-miR-19a | Up | 1.7E-14 | 13q31.3 | 13: 92003145-92003226 [+] | |
| hsa-miR-20a | Up | 4.3E-13 | 13q31.3 | 13: 92003319-92003389 [+] | |
| hsa-miR-19b | Up | 1.8E-14 | 13q31.3 | 13: 92003446-92003532 [+] | |
|  |  |  | Xq26.2 | X: 133303701-133303796 [-] | |
| hsa-miR-342-3p | Up | 1.6E-13 | 14q32.2 | 14: 100575992-100576090 [+] | |
| hsa-miR-411 | Up | 2.1E-05 | 14q32.31 | 14: 101489662-101489757 [+] | |
| **hsa-miR-376a** | Up | 2.9E-09 | 14q32.31 | 14: 101507119-101507186 [+] | |
|  |  |  | 14q32.31 | 14: 101506406-101506485 [+] | |
| hsa-miR-487b^d^ | Up | 0.00012 | 14q32.31 | 14: 101512792-101512875 [+] | |
| hsa-miR-382 | Up | 1.4E-06 | 14q32.31 | 14: 101520643-101520718 [+] | |
| hsa-miR-134^d^ | Up | 4E-05 | 14q32.31 | 14: 101521024-101521096 [+] | |
| hsa-miR-365 | Up | 1E-07 | 16p13.12 | 16: 14403142-14403228 [+] | |
|  |  |  | 17q11.2 | 17: 29902430-29902540 [+] | |
| **hsa-miR-140** | Up | 2.5E-13 | 16q22.1 | 16: 69966984-69967083 [+] | |
| hsa-miR-301a | Up | 1.3E-12 | 17q22 | 17: 57228497-57228582 [+] | |
| hsa-miR-22 | Up | 5.9E-10 | 17p13.3 | 17: 1617197-1617281 [-] | |
| hsa-miR-132 | Up | 2.5E-07 | 17p13.3 | 17: 1953202-1953302 [-] | |
| hsa-miR-152^e^ | Up | 1.6E-06 | 17q21.32 | 17: 46114527-46114613 [-] | |
| hsa-miR-142-3p | Up | 1.4E-14 | 17q22 | 17: 56408593-56408679 [-] | |
| hsa-miR-142-5p | Up | 5.4E-14 | 17q22 | 17: 56408593-56408679 [-] | |
| hsa-miR-21 | Up | 2.7E-14 | 17q23.1 | 17: 57918627-57918698 [+] | |
| hsa-miR-195 | Up | 2.9E-06 | 17p13.1 | 17: 6920934-6921020 [-] | |
| hsa-miR-642a | Up | 5.1E-08 | 19q13.32 | 19: 46178186-46178282 [+] | |
| hsa-miR-199a* | Up | 1.6E-06 | 19p13.2 | 19: 10928102-10928172 [-] | |
|  |  |  | 1q24.3 | 1: 172113675-172113784 [-] | |
| hsa-miR-27a | Up | 4.9E-13 | 19p13.13 | 19: 13947254-13947331 [-] | |
| hsa-miR-23a | Up | 5.9E-12 | 19p13.13 | 19: 13947401-13947473 [-] | |
| hsa-miR-181d | Up | 4.9E-13 | 19p13.13 | 19: 13985689-13985825 [+] | |
| hsa-miR-330 | Up | 1E-08 | 19q13.32 | 19: 46142252-46142345 [-] | |
| hsa-miR-99b^d^ | Up | 7E-12 | 19q13.41 | 19: 52195865-52195934 [+] | |
| hsa-miR-125a | Up | 3E-12 | 19q13.41 | 19: 52196507-52196592 [+] | |
| hsa-miR-26b | Up | 1.4E-14 | 2q35 | 2: 219267369-219267445 [+] | |
| hsa-miR-155 | Up | 5.4E-14 | 21q21.3 | 21: 26946292-26946356 [+] | |
| hsa-miR-130b | Up | 1.2E-08 | 22 | 22: 22007593-22007674 [+] | |
| hsa-let-7b | Up | 1.5E-07 | 22q13.31 | 22: 46509566-46509648 [+] | |
| hsa-miR-374a | Up | 2.9E-14 | Xq13.2 | X: 73507121-73507192 [+] | |
| hsa-miR-224^d^ | Up | 8.5E-07 | Xq28 | X: 151127050-151127130 [-] | |
| hsa-miR-221 | Up | 4.9E-13 | Xp11.3 | X: 45605585-45605694 [-] | |
| hsa-miR-222 | Up | 2.2E-14 | Xp11.3 | X: 45606421-45606530 [-] | |
| hsa-miR-660 | Up | 1.1E-12 | Xp11.23 | X: 49777849-49777945 [+] | |
| hsa-miR-98 | Up | 6.2E-05 | Xp11.22 | X: 53583184-53583302 [-] | |
| hsa-miR-223 | Up | 1.6E-14 | Xq12 | X: 65238712-65238821 [+] | |
| hsa-miR-361 | Up | 1.4E-10 | Xq21.2 | X: 85158641-85158712 [-] | |
| hsa-miR-565 | Up | 8.4E-12 | - | N/A | |

^a^ The bold miRNA was associated with aging.

^b^ p<0.00024 based on the Wilcoxon rank-sum test with Bonferroni correction

^c^ [+]: forward strands; [-]: reverse strand

^d^ Reported to be down-regulated in schizophrenia.

^e^ Reported to be associated with bronchopulmonary dysplasia.

**Table S6**. Distribution of chromosomal locations for detectable miRNAs.

|  | Detectable | Age-constant | Age-limited expression | |  | Age-related modulation | |
| --- | --- | --- | --- | --- | --- | --- | --- |
|  | miRNA  (N^a^ = 264) | expression  (N^a^ = 113) | Preterm infants only  (N^a^ = 1) | Adults only  (N^a^ = 31) |  | Down-regulated  (N^a^ = 22) | Up-regulated  (N^a^ = 96) |
| Chrom. # | n (%) | n (%) | n (%) | n (%) |  | n (%) | n (%) |
| 1 | 22 (8.3) | 11 (9.7) | 0 (0.0) | 3 (9.7) |  | 0 (0.0) | 8 (8.3) |
| 2 | 4 (1.5) | 1 (0.9) | 0 (0.0) | 2 (6.5) |  | 0 (0.0) | 1 (1.0) |
| 3 | 11 (4.2) | 6 (5.3) | 0 (0.0) | 1 (3.2) |  | 1 (4.5) | 3 (3.1) |
| 4 | 5 (1.9) | 3 (2.7) | 0 (0.0) | 0 (0.0) |  | 0 (0.0) | 2 (2.1) |
| 5 | 14 (5.3) | 9 (8.0) | 0 (0.0) | 2 (6.5) |  | 0 (0.0) | 3 (3.1) |
| 6 | 7 (2.7) | 2 (1.8) | 0 (0.0) | 3 (9.7) |  | 1 (4.5) | 1 (1.0) |
| 7 | 15 (5.7) | 4 (3.5) | 0 (0.0) | 1 (3.2) |  | 3 (13.6) | 7 (7.3) |
| 8 | 8 (3) | 2 (1.8) | 0 (0.0) | 1 (3.2) |  | 3 (13.6) | 2 (2.1) |
| 9 | 18 (6.8) | 3 (2.7) | 0 (0.0) | 1 (3.2) |  | 0 (0.0) | 14 (14.6) |
| 10 | 5 (1.9) | 3 (2.7) | 0 (0.0) | 0 (0.0) |  | 0 (0.0) | 2 (2.1) |
| 11 | 9 (3.4) | 5 (4.4) | 0 (0.0) | 0 (0.0) |  | 1 (4.5) | 3 (3.1) |
| 12 | 10 (3.8) | 5 (4.4) | 0 (0.0) | 1 (3.2) |  | 1 (4.5) | 3 (3.1) |
| 13 | 9 (3.4) | 3 (2.7) | 0 (0.0) | 0 (0.0) |  | 1 (4.5) | 5 (5.2) |
| 14 | 34 (12.9) | 23 (20.4) | 0 (0.0) | 2 (6.5) |  | 2 (8.7) | 7 (7.3) |
| 15 | 6 (2.3) | 5 (4.4) | 0 (0.0) | 1 (3.2) |  | 0 (0) | 0 (0.0) |
| 16 | 7 (2.7) | 2 (1.8) | 0 (0.0) | 0 (0.0) |  | 2 (9.1) | 3 (3.1) |
| 17 | 18 (6.8) | 6 (5.3) | 0 (0.0) | 2 (6.5) |  | 2 (9.1) | 8 (8.3) |
| 18 | 3 (1.1) | 2 (1.8) | 0 (0.0) | 1 (3.2) |  | 0 (0.0) | 0 (0.0) |
| 19 | 13 (4.9) | 3 (2.7) | 0 (0.0) | 0 (0.0) |  | 0 (0.0) | 10 (10.4) |
| 20 | 4 (1.5) | 2 (1.8) | 0 (0.0) | 1 (3.2) |  | 1 (4.5) | 0 (0.0) |
| 21 | 4 (1.5) | 2 (1.8) | 0 (0.0) | 0 (0.0) |  | 1 (4.5) | 1 (1.0) |
| 22 | 5 (1.9) | 1 (0.9) | 0 (0.0) | 0 (0.0) |  | 1 (4.5) | 3 (3.1) |
| X | 33 (12.5) | 10 (8.8) | 1 (100) | 9 (29.0) |  | 3 (13.6) | 10 (10.4) |

^a^Number of chromosomal locations for miRNAs; a miRNA might has more than one coordinate chromosomal location

**Table S7.** The detailed chromosomal loci of miRNAs located in four chromosomes that showed a higher proportion for a certain class of miRNAs than the averaged ones as shown in Table S6.

|  | Detectable | Age-constant | Age-limited expression | |  | Age-related modulation | |
| --- | --- | --- | --- | --- | --- | --- | --- |
|  | miRNA  (N^a^ = 264) | expression  (N^a^ = 113) | Preterm infants only  (N^a^ = 1) | Adults only  (N^a^ = 31) |  | Down-regulated  (N^a^ = 22) | Up-regulated  (N^a^ = 96) |
| Chrom. # | n (%) | n (%) | n (%) | n (%) |  | n (%) | n (%) |
| 9 | 18 (6.8) | 3 (2.7) | 0 (0.0) | 1 (3.2) |  | 0 (0.0) | **14 (14.6)** |
| 9q21.12 | 1 | 1 | 0 | 0 |  | 0 | 0 |
| 9q21.3 | 3 | 1 | 0 | 0 |  | 0 | 2 |
| 9q22.32 | 6 | 0 | 0 | 0 |  | 0 | **6** |
| 9q24.1 | 1 | 0 | 0 | 0 |  | 0 | 1 |
| 9q31.3 | 1 | 0 | 0 | 0 |  | 0 | 1 |
| 9q33.3 | 2 | 0 | 0 | 0 |  | 0 | 2 |
| 9q34.11 | 2 | 1 | 0 | 1 |  | 0 | 0 |
| 9q34.3 | 2 | 0 | 0 | 0 |  | 0 | 2 |
| 14 | 34 (12.9) | **23 (20.4)** | 0 (0.0) | 2 (6.5) |  | 2 (8.7) | 7 (7.3) |
| 14q12 | 1 | 1 | 0 | 0 |  | 0 | 0 |
| 14q32.2 | 7 | 4 | 0 | 0 |  | 2 | 1 |
| 14q32.31 | 25 | **17** | 0 | 2 |  | 0 | 6 |
| 14q32.33 | 1 | 1 | 0 | 0 |  | 0 | 0 |
| 19 | 13 (4.9) | 3 (2.7) | 0 (0.0) | 0 (0.0) |  | 0 (0.0) | **10 (10.4)** |
| 19p13.13 | 5 | 1 | 0 | 0 |  | 0 | 4 |
| 19p13.2 | 2 | 1 | 0 | 0 |  | 0 | 1 |
| 19p13.3 | 1 | 0 | 0 | 0 |  | 0 | 1 |
| 19q13.32 | 2 | 0 | 0 | 0 |  | 0 | 2 |
| 19q13.41 | 2 | 1 | 0 | 0 |  | 0 | 2 |
| X | 33 (12.5) | 10 (8.8) | 1 (100) | **9 (29.0)** |  | 3 (13.6) | 10 (10.4) |
| Xp11.2 | 8 | 3 | 0 | 0 |  | 2 | 3 |
| Xp11.3 | 2 | 0 | 0 | 0 |  | 0 | 2 |
| Xp22.31 | 1 | 0 | 0 | 1 |  | 0 | 0 |
| Xq12 | 1 | 0 | 0 | 0 |  | 0 | 1 |
| Xq13.2 | 2 | 1 | 0 | 0 |  | 0 | 1 |
| Xq21 | 2 | 0 | 1 | 0 |  | 0 | 1 |
| Xq23 | 1 | 0 | 0 | 1 |  | 0 | 0 |
| Xq26.2 | 4 | 2 | 0 | 0 |  | 1 | 1 |
| Xq26.3 | 5 | 2 | 0 | 3 |  | 0 | 0 |
| Xq27.3 | 4 | 0 | 0 | 4 |  | 0 | 0 |
| Xq28 | 3 | 2 | 0 | 0 |  | 0 | 1 |

**Table S8.** Top five associated canonical pathways for predicted miR-target genes of the miRNA that were clustered on14q32.31 and 9q22.

| Canonical pathways | Pathway Category | Numbers of target genes^a^ | Numbers of miRNAs^b^ | p-value^c^ | Ratio^d^  (%) |
| --- | --- | --- | --- | --- | --- |
| Predicted miR-target genes (n = 3209) of age-constant expression miRNAs clustered on 14q32.31 | | | | | |
| Wnt/β-catenin Signaling | Organismal growth and development, cancer | 59 | 11 | 1.7E-14 | 66/175 (0.38) |
| Mouse embryonic stem cell pluripotency | Organismal growth and development, cellular growth, proliferation and development | 38 | 8 | 3.4E-14 | 45/99 (0.46) |
| Factors promoting cardiogenesis in Vertebrates | Organismal growth and development, cardiovascular signaling | 35 | 7 | 1.7E-13 | 42/95 (0.44) |
| Molecular mechanisms of cancer | Disease-specific pathways, cancer | 96 | 9 | 6.8E-13 | 103/381 (0.27) |
| Regulation of the epithelial-mesenchymal transition pathway | Organismal growth and development | 58 | 7 | 9.8E-13 | 65/192 (0.34) |
| Predicted miR-target genes (n = 1793) of age-related up-regulation in adults miRNAs clustered on 9q22.32 | | | | | |
| Molecular mechanisms of cancer | Cancer, disease-specific pathways | 74 | 2 | 6.4E-15 | 74/381 (0.20) |
| Estrogen-mediated S-phase entry | Cell cycle regulation, nuclear receptor signaling | 14 | 2 | 4.0E-10 | 14/28 (0.50) |
| PTEN signaling | Cancer, apoptosis | 31 | 2 | 2.9E-09 | 31/133 (0.23) |
| Glioma signaling | Cancer, disease-specific pathways | 27 | 2 | 1.3E-08 | 27/112 (0.24) |
| Chronic myeloid leukemia signaling | Cancer, disease-specific pathways | 26 | 2 | 1.4E-08 | 26/105 (0.25) |
| ^a^ Among all submitted predicted miR-target genes, the numbers of target genes were involved in the pathway.  ^b^ Numbers of miRNA which regulates miR-target genes that involved in the pathway  ^c^ Based on Fisher's exact test determining the probability that the association between the genes in the data set and the canonical pathway is due to chance alone  ^d^A ratio of the number of genes from the data set that map to the pathway divided by the total number of genes that map to the canonical pathway | | | | | |

**Table S9**. The target genes derived from the top five associated canonical pathways for the age-constant expression miRNAs clustered on 14q32.31.

| Pathway | Target gene | microRNA | Prediction databases | Confidence |
| --- | --- | --- | --- | --- |
| I: Wnt/β-catenin Signaling | ACVR1 | hsa-miR-381 | TargetScan Human | High (predicted) |
|  | ACVR1C | hsa-miR-410 | TargetScan Human | High (predicted) |
|  |  | hsa-miR-494 | TargetScan Human | High (predicted) |
|  | ACVR2B | hsa-miR-410 | TargetScan Human | High (predicted) |
|  |  | hsa-miR-381 | TargetScan Human | High (predicted) |
|  | AKT2 | hsa-miR-329 | TargetScan Human | High (predicted) |
|  | AKT3 | hsa-miR-654 | TargetScan Human | High (predicted) |
|  | APC | hsa-miR-381 | TargetScan Human | High (predicted) |
|  |  | hsa-miR-494 | TargetScan Human | High (predicted) |
|  | APC2 | hsa-miR-654 | TargetScan Human | High (predicted) |
|  | APPL1 | hsa-miR-381 | TargetScan Human | High (predicted) |
|  | BMPR2 | hsa-miR-329 | TargetScan Human | High (predicted) |
|  |  | hsa-miR-381 | TargetScan Human | High (predicted) |
|  |  | hsa-miR-494 | TargetScan Human | High (predicted) |
|  | CD44 | hsa-miR-381 | TargetScan Human | High (predicted) |
|  | CDH2 | hsa-miR-496 | TargetScan Human | High (predicted) |
|  | CREBBP | hsa-miR-410 | TargetScan Human | High (predicted) |
|  |  | hsa-miR-381 | TargetScan Human | High (predicted) |
|  | CSNK1G1 | hsa-miR-410 | TargetScan Human | High (predicted) |
|  |  | hsa-miR-494 | TargetScan Human | High (predicted) |
|  | CSNK1G3 | hsa-miR-329 | TargetScan Human | High (predicted) |
|  | CSNK2A1 | hsa-miR-329 | TargetScan Human | High (predicted) |
|  |  | hsa-miR-485-5p | TargetScan Human | High (predicted) |
|  | CSNK2A2 | hsa-miR-654 | TargetScan Human | High (predicted) |
|  | CTNNB1 | hsa-miR-381 | TargetScan Human | High (predicted) |
|  | DKK3 | hsa-miR-381 | TargetScan Human | High (predicted) |
|  | DVL2 | hsa-miR-381 | TargetScan Human | High (predicted) |
|  | DVL3 | hsa-miR-485-5p | TargetScan Human | High (predicted) |
|  | FZD1 | hsa-miR-410 | TargetScan Human | High (predicted) |
|  | FZD2 | hsa-miR-410 | TargetScan Human | High (predicted) |
|  |  | hsa-miR-494 | TargetScan Human | High (predicted) |
|  | FZD3 | hsa-miR-381 | TargetScan Human | High (predicted) |
|  | FZD4 | hsa-miR-485-5p | TargetScan Human | High (predicted) |
|  |  | hsa-miR-496 | TargetScan Human | High (predicted) |
|  | FZD5 | hsa-miR-410 | TargetScan Human | High (predicted) |
|  | FZD8 | hsa-miR-410 | TargetScan Human | High (predicted) |
|  | GSK3A | hsa-miR-485-5p | TargetScan Human | High (predicted) |
|  | GSK3B | hsa-miR-410 | TargetScan Human | High (predicted) |
|  |  | hsa-miR-381 | TargetScan Human | High (predicted) |
|  | HDAC1 | hsa-miR-410 | TargetScan Human | High (predicted) |
|  | LEF1 | hsa-miR-381 | TargetScan Human | High (predicted) |
|  | LRP6 | hsa-miR-410 | TargetScan Human | High (predicted) |
|  |  | hsa-miR-381 | TargetScan Human | High (predicted) |
|  | MDM2 | hsa-miR-379-5p | TargetScan Human | High (predicted) |
|  |  | hsa-miR-409-5p | TargetScan Human | High (predicted) |
|  |  | hsa-miR-485-5p | TargetScan Human | High (predicted) |
|  |  | hsa-miR-494 | TargetScan Human | High (predicted) |
|  | NLK | hsa-miR-381 | TargetScan Human | High (predicted) |
|  | NR5A2 | hsa-miR-381 | TargetScan Human | High (predicted) |
|  | PPP2R2B | hsa-miR-494 | TargetScan Human | High (predicted) |
|  | PPP2R2C | hsa-miR-329 | TargetScan Human | High (predicted) |
|  |  | hsa-miR-381 | TargetScan Human | High (predicted) |
|  | PPP2R5E | hsa-miR-494 | TargetScan Human | High (predicted) |
|  | RARB | hsa-miR-379-5p | TargetScan Human | High (predicted) |
|  | SFRP2 | hsa-miR-381 | TargetScan Human | High (predicted) |
|  | SMO | hsa-miR-381 | TargetScan Human | High (predicted) |
|  | SOX11 | hsa-miR-381 | TargetScan Human | High (predicted) |
|  | SOX17 | hsa-miR-329 | TargetScan Human | High (predicted) |
|  | SOX2 | hsa-miR-381 | TargetScan Human | High (predicted) |
|  | SOX4 | hsa-miR-381 | TargetScan Human | High (predicted) |
|  | SOX5 | hsa-miR-485-5p | TargetScan Human | High (predicted) |
|  | SOX6 | hsa-miR-412 | TargetScan Human | High (predicted) |
|  | SOX7 | hsa-miR-410 | TargetScan Human | High (predicted) |
|  |  | hsa-miR-485-3p | TargetScan Human | High (predicted) |
|  |  | hsa-miR-494 | TargetScan Human | High (predicted) |
|  | SOX9 | hsa-miR-381 | TargetScan Human | High (predicted) |
|  | TAB1 | hsa-miR-654 | TargetScan Human | High (predicted) |
|  | TCF3 | hsa-miR-381 | TargetScan Human | High (predicted) |
|  | TCF4 | hsa-miR-410 | TargetScan Human | High (predicted) |
|  | TCF7 | hsa-miR-485-5p | TargetScan Human | High (predicted) |
|  | TCF7L1 | hsa-miR-329 | TargetScan Human | High (predicted) |
|  | TCF7L2 | hsa-miR-410 | TargetScan Human | High (predicted) |
|  |  | hsa-miR-494 | TargetScan Human | High (predicted) |
|  | TGFB3 | hsa-miR-381 | TargetScan Human | High (predicted) |
|  | TGFBR1 | hsa-miR-381 | TargetScan Human | High (predicted) |
|  | TGFBR2 | hsa-miR-410 | TargetScan Human | High (predicted) |
|  |  | hsa-miR-496 | TargetScan Human | High (predicted) |
|  | TLE1 | hsa-miR-381 | TargetScan Human | High (predicted) |
|  | TP53 | hsa-miR-485-5p | TargetScan Human | High (predicted), Moderate (predicted) |
| II: Mouse Embryonic Stem Cell Pluripotency | AKT2 | hsa-miR-329 | TargetScan Human | High (predicted) |
|  | AKT3 | hsa-miR-654 | TargetScan Human | High (predicted) |
|  | APC | hsa-miR-381 | TargetScan Human | High (predicted) |
|  |  | hsa-miR-494 | TargetScan Human | High (predicted) |
|  | BMPR2 | hsa-miR-329 | TargetScan Human | High (predicted) |
|  |  | hsa-miR-381 | TargetScan Human | High (predicted) |
|  |  | hsa-miR-494 | TargetScan Human | High (predicted) |
|  | CREBBP | hsa-miR-410 | TargetScan Human | High (predicted) |
|  |  | hsa-miR-381 | TargetScan Human | High (predicted) |
|  | CTNNB1 | hsa-miR-381 | TargetScan Human | High (predicted) |
|  | DVL2 | hsa-miR-381 | TargetScan Human | High (predicted) |
|  | DVL3 | hsa-miR-485-5p | TargetScan Human | High (predicted) |
|  | FOXD3 | hsa-miR-410 | TargetScan Human | High (predicted) |
|  | FZD1 | hsa-miR-410 | TargetScan Human | High (predicted) |
|  | FZD2 | hsa-miR-410 | TargetScan Human | High (predicted) |
|  |  | hsa-miR-494 | TargetScan Human | High (predicted) |
|  | FZD3 | hsa-miR-381 | TargetScan Human | High (predicted) |
|  | FZD4 | hsa-miR-485-5p | TargetScan Human | High (predicted) |
|  |  | hsa-miR-496 | TargetScan Human | High (predicted) |
|  | FZD5 | hsa-miR-410 | TargetScan Human | High (predicted) |
|  | FZD8 | hsa-miR-410 | TargetScan Human | High (predicted) |
|  | GRB2 | hsa-miR-329 | TargetScan Human | High (predicted) |
|  | GSK3B | hsa-miR-410 | TargetScan Human | High (predicted) |
|  |  | hsa-miR-381 | TargetScan Human | High (predicted) |
|  | ID1 | hsa-miR-381 | TargetScan Human | High (predicted) |
|  | ID2 | hsa-miR-381 | TargetScan Human | High (predicted) |
|  | ID4 | hsa-miR-369-5p | TargetScan Human | High (predicted) |
|  |  | hsa-miR-381 | TargetScan Human | High (predicted) |
|  | KRAS | hsa-miR-381 | TargetScan Human | High (predicted) |
|  | LEF1 | hsa-miR-381 | TargetScan Human | High (predicted) |
|  | LIF | hsa-miR-494 | TargetScan Human | High (predicted) |
|  | MAPK1 | hsa-miR-410 | TargetScan Human | High (predicted) |
|  | MAPK14 | hsa-miR-654 | TargetScan Human | High (predicted) |
|  | PIK3R3 | hsa-miR-494 | TargetScan Human | High (predicted) |
|  | PIK3R6 | hsa-miR-654 | TargetScan Human | High (predicted) |
|  | PTPN11 | hsa-miR-494 | TargetScan Human | High (predicted) |
|  | RRAS2 | hsa-miR-381 | TargetScan Human | High (predicted) |
|  | SMAD4 | hsa-miR-381 | TargetScan Human | High (predicted) |
|  | SMAD9 | hsa-miR-494 | TargetScan Human | High (predicted) |
|  | SMO | hsa-miR-381 | TargetScan Human | High (predicted) |
|  | SOS1 | hsa-miR-410 | TargetScan Human | High (predicted) |
|  | SOX2 | hsa-miR-381 | TargetScan Human | High (predicted) |
|  | STAT3 | hsa-miR-410 | TargetScan Human | High (predicted) |
|  | TAB1 | hsa-miR-654 | TargetScan Human | High (predicted) |
|  | TCF3 | hsa-miR-381 | TargetScan Human | High (predicted) |
|  | TCF4 | hsa-miR-410 | TargetScan Human | High (predicted) |
| III: Factors Promoting Cardiogenesis in Vertebrates | ACVR1 | hsa-miR-381 | TargetScan Human | High (predicted) |
|  | ACVR1C | hsa-miR-410 | TargetScan Human | High (predicted) |
|  |  | hsa-miR-494 | TargetScan Human | High (predicted) |
|  | ACVR2B | hsa-miR-410 | TargetScan Human | High (predicted) |
|  |  | hsa-miR-381 | TargetScan Human | High (predicted) |
|  | APC | hsa-miR-381 | TargetScan Human | High (predicted) |
|  |  | hsa-miR-494 | TargetScan Human | High (predicted) |
|  | BMP1 | hsa-miR-654 | TargetScan Human | High (predicted) |
|  | BMP8A | hsa-miR-485-5p | TargetScan Human | High (predicted) |
|  |  | hsa-miR-654 | TargetScan Human | High (predicted) |
|  | BMPR1B | hsa-miR-329 | TargetScan Human | High (predicted) |
|  |  | hsa-miR-381 | TargetScan Human | High (predicted) |
|  | BMPR2 | hsa-miR-329 | TargetScan Human | High (predicted) |
|  |  | hsa-miR-381 | TargetScan Human | High (predicted) |
|  |  | hsa-miR-494 | TargetScan Human | High (predicted) |
|  | CCNE2 | hsa-miR-485-5p | TargetScan Human | High (predicted) |
|  | CDC6 | hsa-miR-654 | TargetScan Human | High (predicted) |
|  | CTNNB1 | hsa-miR-381 | TargetScan Human | High (predicted) |
|  | FZD1 | hsa-miR-410 | TargetScan Human | High (predicted) |
|  | FZD2 | hsa-miR-410 | TargetScan Human | High (predicted) |
|  |  | hsa-miR-494 | TargetScan Human | High (predicted) |
|  | FZD3 | hsa-miR-381 | TargetScan Human | High (predicted) |
|  | FZD4 | hsa-miR-485-5p | TargetScan Human | High (predicted) |
|  |  | hsa-miR-496 | TargetScan Human | High (predicted) |
|  | FZD5 | hsa-miR-410 | TargetScan Human | High (predicted) |
|  | FZD8 | hsa-miR-410 | TargetScan Human | High (predicted) |
|  | GATA4 | hsa-miR-654 | TargetScan Human | High (predicted) |
|  | GSK3B | hsa-miR-410 | TargetScan Human | High (predicted) |
|  |  | hsa-miR-381 | TargetScan Human | High (predicted) |
|  | LEF1 | hsa-miR-381 | TargetScan Human | High (predicted) |
|  | LRP6 | hsa-miR-410 | TargetScan Human | High (predicted) |
|  |  | hsa-miR-381 | TargetScan Human | High (predicted) |
|  | MAPK14 | hsa-miR-654 | TargetScan Human | High (predicted) |
|  | MEF2C | hsa-miR-410 | TargetScan Human | High (predicted) |
|  |  | hsa-miR-381 | TargetScan Human | High (predicted) |
|  | NODAL | hsa-miR-654 | TargetScan Human | High (predicted) |
|  | NOG | hsa-miR-329 | TargetScan Human | High (predicted) |
|  | PRKCA | hsa-miR-329 | TargetScan Human | High (predicted) |
|  | PRKCE | hsa-miR-381 | TargetScan Human | High (predicted) |
|  |  | hsa-miR-494 | TargetScan Human | High (predicted) |
|  |  | hsa-miR-496 | TargetScan Human | High (predicted) |
|  | SMAD2 | hsa-miR-329 | TargetScan Human | High (predicted) |
|  |  | hsa-miR-410 | TargetScan Human | High (predicted) |
|  | SMAD4 | hsa-miR-381 | TargetScan Human | High (predicted) |
|  | SMAD9 | hsa-miR-494 | TargetScan Human | High (predicted) |
|  | SMO | hsa-miR-381 | TargetScan Human | High (predicted) |
|  | TBX5 | hsa-miR-329 | TargetScan Human | High (predicted) |
|  |  | hsa-miR-410 | TargetScan Human | High (predicted) |
|  |  | hsa-miR-494 | TargetScan Human | High (predicted) |
|  | TCF3 | hsa-miR-381 | TargetScan Human | High (predicted) |
|  | TCF4 | hsa-miR-410 | TargetScan Human | High (predicted) |
|  | TCF7 | hsa-miR-485-5p | TargetScan Human | High (predicted) |
| IV: Molecular Mechanisms of Cancer | ADCY5 | hsa-miR-494 | TargetScan Human | High (predicted) |
|  | ADCY6 | hsa-miR-494 | TargetScan Human | High (predicted) |
|  | ADCY9 | hsa-miR-329 | TargetScan Human | High (predicted) |
|  | AKT2 | hsa-miR-329 | TargetScan Human | High (predicted) |
|  | AKT3 | hsa-miR-654 | TargetScan Human | High (predicted) |
|  | APAF1 | hsa-miR-381 | TargetScan Human | High (predicted) |
|  | APC | hsa-miR-381 | TargetScan Human | High (predicted) |
|  |  | hsa-miR-494 | TargetScan Human | High (predicted) |
|  | ARHGEF12 | hsa-miR-494 | TargetScan Human | High (predicted) |
|  | ARHGEF15 | hsa-miR-485-5p | TargetScan Human | High (predicted) |
|  | ARHGEF17 | hsa-miR-379-5p | TargetScan Human | High (predicted) |
|  |  | hsa-miR-494 | TargetScan Human | High (predicted) |
|  | ARHGEF19 | hsa-miR-379-5p | TargetScan Human | High (predicted) |
|  | ARHGEF3 | hsa-miR-412 | TargetScan Human | High (predicted) |
|  |  | hsa-miR-381 | TargetScan Human | High (predicted) |
|  | ARHGEF6 | hsa-miR-496 | TargetScan Human | High (predicted) |
|  |  | hsa-miR-409-5p | TargetScan Human | High (predicted) |
|  | BBC3 | hsa-miR-494 | TargetScan Human | High (predicted) |
|  | BCL2 | hsa-miR-410 | TargetScan Human | High (predicted) |
|  | BCL2L11 | hsa-miR-381 | TargetScan Human | High (predicted) |
|  |  | hsa-miR-494 | TargetScan Human | High (predicted) |
|  | BMP1 | hsa-miR-654 | TargetScan Human | High (predicted) |
|  | BMP8A | hsa-miR-654 | TargetScan Human | High (predicted) |
|  |  | hsa-miR-485-5p | TargetScan Human | High (predicted) |
|  | BMPR1B | hsa-miR-329 | TargetScan Human | High (predicted) |
|  |  | hsa-miR-381 | TargetScan Human | High (predicted) |
|  | BMPR2 | hsa-miR-329 | TargetScan Human | High (predicted) |
|  |  | hsa-miR-381 | TargetScan Human | High (predicted) |
|  |  | hsa-miR-494 | TargetScan Human | High (predicted) |
|  | CAMK2A | hsa-miR-654 | TargetScan Human | High (predicted) |
|  |  | hsa-miR-485-5p | TargetScan Human | High (predicted) |
|  | CAMK2D | hsa-miR-494 | TargetScan Human | High (predicted) |
|  | CBL | hsa-miR-410 | TargetScan Human | High (predicted) |
|  |  | hsa-miR-381 | TargetScan Human | High (predicted) |
|  | CCND2 | hsa-miR-381 | TargetScan Human | High (predicted) |
|  |  | hsa-miR-494 | TargetScan Human | High (predicted) |
|  | CCNE2 | hsa-miR-485-5p | TargetScan Human | High (predicted) |
|  | CDC42 | hsa-miR-381 | TargetScan Human | High (predicted) |
|  | CDK6 | hsa-miR-494 | TargetScan Human | High (predicted) |
|  | CDKN1A | hsa-miR-485-5p | TargetScan Human | High (predicted) |
|  | CDKN2D | hsa-miR-329 | TargetScan Human | High (predicted) |
|  | CHEK1 | hsa-miR-654 | TargetScan Human | High (predicted) |
|  | CREBBP | hsa-miR-410 | TargetScan Human | High (predicted) |
|  |  | hsa-miR-381 | TargetScan Human | High (predicted) |
|  | CRK | hsa-miR-381 | TargetScan Human | High (predicted) |
|  | CTNNA2 | hsa-miR-381 | TargetScan Human | High (predicted) |
|  | CTNNB1 | hsa-miR-381 | TargetScan Human | High (predicted) |
|  | CTNND1 | hsa-miR-329 | TargetScan Human | High (predicted) |
|  |  | hsa-miR-410 | TargetScan Human | High (predicted) |
|  | CYCS | hsa-miR-410 | TargetScan Human | High (predicted) |
|  | E2F1 | hsa-miR-329 | TargetScan Human | High (predicted) |
|  | FOXO1 | hsa-miR-381 | TargetScan Human | High (predicted) |
|  | FZD1 | hsa-miR-410 | TargetScan Human | High (predicted) |
|  | FZD2 | hsa-miR-410 | TargetScan Human | High (predicted) |
|  |  | hsa-miR-494 | TargetScan Human | High (predicted) |
|  | FZD3 | hsa-miR-381 | TargetScan Human | High (predicted) |
|  | FZD4 | hsa-miR-496 | TargetScan Human | High (predicted) |
|  |  | hsa-miR-485-5p | TargetScan Human | High (predicted) |
|  | FZD5 | hsa-miR-410 | TargetScan Human | High (predicted) |
|  | FZD8 | hsa-miR-410 | TargetScan Human | High (predicted) |
|  | GAB1 | hsa-miR-410 | TargetScan Human | High (predicted) |
|  | GAB2 | hsa-miR-485-5p | TargetScan Human | High (predicted) |
|  | GNAI3 | hsa-miR-494 | TargetScan Human | High (predicted) |
|  | GNAL | hsa-miR-485-5p | TargetScan Human | High (predicted) |
|  | GRB2 | hsa-miR-329 | TargetScan Human | High (predicted) |
|  | GSK3A | hsa-miR-485-5p | TargetScan Human | High (predicted) |
|  | GSK3B | hsa-miR-410 | TargetScan Human | High (predicted) |
|  |  | hsa-miR-381 | TargetScan Human | High (predicted) |
|  | HHAT | hsa-miR-485-5p | TargetScan Human | High (predicted) |
|  | HIPK2 | hsa-miR-381 | TargetScan Human | High (predicted) |
|  |  | hsa-miR-485-5p | TargetScan Human | High (predicted) |
|  | IRS1 | hsa-miR-410 | TargetScan Human | High (predicted) |
|  |  | hsa-miR-654 | TargetScan Human | High (predicted) |
|  |  | hsa-miR-494 | TargetScan Human | High (predicted) |
|  | KRAS | hsa-miR-381 | TargetScan Human | High (predicted) |
|  | LEF1 | hsa-miR-381 | TargetScan Human | High (predicted) |
|  | LRP6 | hsa-miR-410 | TargetScan Human | High (predicted) |
|  |  | hsa-miR-381 | TargetScan Human | High (predicted) |
|  | MAPK1 | hsa-miR-410 | TargetScan Human | High (predicted) |
|  | MAPK14 | hsa-miR-654 | TargetScan Human | High (predicted) |
|  | MDM2 | hsa-miR-379-5p | TargetScan Human | High (predicted) |
|  |  | hsa-miR-409-5p | TargetScan Human | High (predicted) |
|  |  | hsa-miR-485-5p | TargetScan Human | High (predicted) |
|  |  | hsa-miR-494 | TargetScan Human | High (predicted) |
|  | NAIP | hsa-miR-409-5p | TargetScan Human | High (predicted) |
|  | NFKBIA | hsa-miR-381 | TargetScan Human | High (predicted) |
|  | NLK | hsa-miR-381 | TargetScan Human | High (predicted) |
|  | PAK1 | hsa-miR-485-5p | TargetScan Human | High (predicted) |
|  | PAK4 | hsa-miR-369-5p | TargetScan Human | High (predicted) |
|  |  | hsa-miR-485-5p | TargetScan Human | High (predicted) |
|  | PIK3R3 | hsa-miR-494 | TargetScan Human | High (predicted) |
|  | PIK3R6 | hsa-miR-654 | TargetScan Human | High (predicted) |
|  | PLCB1 | hsa-miR-494 | TargetScan Human | High (predicted) |
|  | PLCB3 | hsa-miR-485-5p | TargetScan Human | High (predicted) |
|  | PRKAR2B | hsa-miR-410 | TargetScan Human | High (predicted) |
|  | PRKCA | hsa-miR-329 | TargetScan Human | High (predicted) |
|  | PRKCE | hsa-miR-496 | TargetScan Human | High (predicted) |
|  |  | hsa-miR-381 | TargetScan Human | High (predicted) |
|  |  | hsa-miR-494 | TargetScan Human | High (predicted) |
|  | PTCH1 | hsa-miR-381 | TargetScan Human | High (predicted) |
|  | PTK2 | hsa-miR-410 | TargetScan Human | High (predicted) |
|  |  | hsa-miR-379-5p | TargetScan Human | High (predicted) |
|  | PTPN11 | hsa-miR-494 | TargetScan Human | High (predicted) |
|  | RALA | hsa-miR-329 | TargetScan Human | High (predicted) |
|  | RAP1B | hsa-miR-494 | TargetScan Human | High (predicted) |
|  | RAP2B | hsa-miR-494 | TargetScan Human | High (predicted) |
|  | RASGRF1 | hsa-miR-494 | TargetScan Human | High (predicted) |
|  | RBPJ | hsa-miR-381 | TargetScan Human | High (predicted) |
|  | RHOA | hsa-miR-654 | TargetScan Human | High (predicted) |
|  | RRAS2 | hsa-miR-381 | TargetScan Human | High (predicted) |
|  | SIN3A | hsa-miR-329 | TargetScan Human | High (predicted) |
|  | SMAD2 | hsa-miR-329 | TargetScan Human | High (predicted) |
|  |  | hsa-miR-410 | TargetScan Human | High (predicted) |
|  | SMAD4 | hsa-miR-381 | TargetScan Human | High (predicted) |
|  | SMAD6 | hsa-miR-410 | TargetScan Human | High (predicted) |
|  | SMAD7 | hsa-miR-410 | TargetScan Human | High (predicted) |
|  | SMAD9 | hsa-miR-494 | TargetScan Human | High (predicted) |
|  | SMO | hsa-miR-381 | TargetScan Human | High (predicted) |
|  | SOS1 | hsa-miR-410 | TargetScan Human | High (predicted) |
|  | SUFU | hsa-miR-485-5p | TargetScan Human | High (predicted) |
|  |  | hsa-miR-494 | TargetScan Human | High (predicted) |
|  | SYNGAP1 | hsa-miR-329 | TargetScan Human | High (predicted) |
|  |  | hsa-miR-410 | TargetScan Human | High (predicted) |
|  |  | hsa-miR-654 | TargetScan Human | High (predicted) |
|  | TAB1 | hsa-miR-654 | TargetScan Human | High (predicted) |
|  | TCF3 | hsa-miR-381 | TargetScan Human | High (predicted) |
|  | TCF4 | hsa-miR-410 | TargetScan Human | High (predicted) |
|  | TGFB3 | hsa-miR-381 | TargetScan Human | High (predicted) |
| V: Regulation of the epithelial-mesenchymal transition pathway | AKT2 | hsa-miR-329 | TargetScan Human | High (predicted) |
|  | AKT3 | hsa-miR-654 | TargetScan Human | High (predicted) |
|  | APC | hsa-miR-381 | TargetScan Human | High (predicted) |
|  |  | hsa-miR-494 | TargetScan Human | High (predicted) |
|  | ARAF | hsa-miR-654 | TargetScan Human | High (predicted) |
|  | CDH2 | hsa-miR-496 | TargetScan Human | High (predicted) |
|  | DVL2 | hsa-miR-381 | TargetScan Human | High (predicted) |
|  | DVL3 | hsa-miR-485-5p | TargetScan Human | High (predicted) |
|  | EGF | hsa-miR-485-5p | TargetScan Human | High (predicted) |
|  | ETS1 | hsa-miR-410 | TargetScan Human | High (predicted) |
|  |  | hsa-miR-381 | TargetScan Human | High (predicted) |
|  | FGF1 | hsa-miR-381 | TargetScan Human | High (predicted) |
|  | FGF16 | hsa-miR-494 | miRecords | Experimentally Observed |
|  | FGF19 | hsa-miR-654 | TargetScan Human | High (predicted) |
|  | FGF7 | hsa-miR-410 | TargetScan Human | High (predicted) |
|  |  | hsa-miR-381 | TargetScan Human | High (predicted) |
|  |  | hsa-miR-494 | TargetScan Human | High (predicted) |
|  | FGF9 | hsa-miR-410 | TargetScan Human | High (predicted) |
|  |  | hsa-miR-494 | TargetScan Human | High (predicted) |
|  | FGFR2 | hsa-miR-410 | TargetScan Human | High (predicted) |
|  |  | hsa-miR-381 | TargetScan Human | High (predicted) |
|  |  | hsa-miR-494 | TargetScan Human | High (predicted) |
|  | FRS2 | hsa-miR-381 | TargetScan Human | High (predicted) |
|  | FZD1 | hsa-miR-410 | TargetScan Human | High (predicted) |
|  | FZD2 | hsa-miR-410 | TargetScan Human | High (predicted) |
|  |  | hsa-miR-494 | TargetScan Human | High (predicted) |
|  | FZD3 | hsa-miR-381 | TargetScan Human | High (predicted) |
|  | FZD4 | hsa-miR-485-5p | TargetScan Human | High (predicted) |
|  |  | hsa-miR-496 | TargetScan Human | High (predicted) |
|  | FZD5 | hsa-miR-410 | TargetScan Human | High (predicted) |
|  | FZD8 | hsa-miR-410 | TargetScan Human | High (predicted) |
|  | GAB1 | hsa-miR-410 | TargetScan Human | High (predicted) |
|  | GRB2 | hsa-miR-329 | TargetScan Human | High (predicted) |
|  | GSK3B | hsa-miR-410 | TargetScan Human | High (predicted) |
|  |  | hsa-miR-381 | TargetScan Human | High (predicted) |
|  | HMGA2 | hsa-miR-410 | TargetScan Human | High (predicted) |
|  |  | hsa-miR-485-5p | TargetScan Human | High (predicted) |
|  | ID2 | hsa-miR-381 | TargetScan Human | High (predicted) |
|  | JAG1 | hsa-miR-381 | TargetScan Human | High (predicted) |
|  | JAG2 | hsa-miR-381 | TargetScan Human | High (predicted) |
|  | KRAS | hsa-miR-381 | TargetScan Human | High (predicted) |
|  | LEF1 | hsa-miR-381 | TargetScan Human | High (predicted) |
|  | MAP2K7 | hsa-miR-654 | TargetScan Human | High (predicted) |
|  | MAPK1 | hsa-miR-410 | TargetScan Human | High (predicted) |
|  | PARD6B | hsa-miR-381 | TargetScan Human | High (predicted) |
|  | PARD6G | hsa-miR-410 | TargetScan Human | High (predicted) |
|  | PDGFRB | hsa-miR-654 | TargetScan Human | High (predicted) |
|  | PIK3R3 | hsa-miR-494 | TargetScan Human | High (predicted) |
|  | PIK3R6 | hsa-miR-654 | TargetScan Human | High (predicted) |
|  | PTPN11 | hsa-miR-494 | TargetScan Human | High (predicted) |
|  | RBPJ | hsa-miR-381 | TargetScan Human | High (predicted) |
|  | RHOA | hsa-miR-654 | TargetScan Human | High (predicted) |
|  | RRAS2 | hsa-miR-381 | TargetScan Human | High (predicted) |
|  | SMAD2 | hsa-miR-329 | TargetScan Human | High (predicted) |
|  |  | hsa-miR-410 | TargetScan Human | High (predicted) |
|  | SMAD4 | hsa-miR-381 | TargetScan Human | High (predicted) |
|  | SMO | hsa-miR-381 | TargetScan Human | High (predicted) |
|  | SNAI1 | hsa-miR-410 | TargetScan Human | High (predicted) |
|  |  | hsa-miR-381 | TargetScan Human | High (predicted) |
|  | SOS1 | hsa-miR-410 | TargetScan Human | High (predicted) |
|  | STAT3 | hsa-miR-410 | TargetScan Human | High (predicted) |
|  | TCF3 | hsa-miR-381 | TargetScan Human | High (predicted) |
|  | TCF4 | hsa-miR-410 | TargetScan Human | High (predicted) |
|  | TCF7 | hsa-miR-485-5p | TargetScan Human | High (predicted) |
|  | TCF7L1 | hsa-miR-329 | TargetScan Human | High (predicted) |
|  | TCF7L2 | hsa-miR-410 | TargetScan Human | High (predicted) |
|  |  | hsa-miR-494 | TargetScan Human | High (predicted) |
|  | TGFB3 | hsa-miR-381 | TargetScan Human | High (predicted) |
|  | TGFBR1 | hsa-miR-381 | TargetScan Human | High (predicted) |
|  | TGFBR2 | hsa-miR-410 | TargetScan Human | High (predicted) |
|  |  | hsa-miR-496 | TargetScan Human | High (predicted) |
|  | TWIST1 | hsa-miR-381 | TargetScan Human | High (predicted) |
|  | TYK2 | hsa-miR-654 | TargetScan Human | High (predicted) |

**Table S10**. The target genes derived from the top five associated canonical pathways for the the age-related up-regulation expression in adults miRNAs clustered on 9q22.32.

| Pathway | Target gene | microRNA | Prediction databases | Confidence |
| --- | --- | --- | --- | --- |
| I: Molecular Mechanisms of Cancer | ADCY9 | hsa-let-7f | TargetScan Human | High (predicted) |
|  |  | hsa-miR-24 | TargetScan Human | High (predicted) |
|  | AKT2 | hsa-let-7f | TargetScan Human | High (predicted) |
|  | ARHGEF15 | hsa-let-7f | TargetScan Human | High (predicted) |
|  | ARHGEF7 | hsa-let-7f | TargetScan Human | High (predicted) |
|  | BBC3 | hsa-miR-24 | TargetScan Human | High (predicted) |
|  | BCL2L1 | hsa-let-7f | Ingenuity Expert Findings, TargetScan Human | Experimentally Observed, High (predicted) |
|  | BCL2L11 | hsa-miR-24 | TargetScan Human | High (predicted) |
|  | BMPR1B | hsa-miR-24 | TargetScan Human | High (predicted) |
|  | BMPR2 | hsa-miR-24 | TargetScan Human | High (predicted) |
|  | BRCA1 | hsa-miR-24 | miRecords | Experimentally Observed |
|  | CAMK2B | hsa-miR-24 | TargetScan Human | High (predicted) |
|  | CAMK2D | hsa-miR-24 | TargetScan Human | High (predicted) |
|  | CASP3 | hsa-let-7f | TargetScan Human, miRecords | Experimentally Observed, High (predicted) |
|  | CBL | hsa-let-7f | TargetScan Human | High (predicted) |
|  | CCND1 | hsa-let-7f | Ingenuity Expert Findings, TarBase, TargetScan Human, miRecords | Experimentally Observed, High (predicted) |
|  | CCND2 | hsa-let-7f | TargetScan Human | High (predicted) |
|  | CDC25A | hsa-let-7f | TargetScan Human, miRecords | Experimentally Observed, High (predicted) |
|  | CDK4 | hsa-miR-24 | miRecords | Experimentally Observed |
|  | CDK6 | hsa-let-7f | TargetScan Human, miRecords | Experimentally Observed, High (predicted) |
|  | CDKN1A | hsa-let-7f | TargetScan Human | High (predicted) |
|  | CDKN1B | hsa-miR-24 | TargetScan Human | High (predicted) |
|  | CDKN2A | hsa-miR-24 | TarBase, miRecords | Experimentally Observed |
|  | CFLAR | hsa-miR-24 | TargetScan Human | High (predicted) |
|  | E2F2 | hsa-let-7f | TargetScan Human | High (predicted) |
|  |  | hsa-miR-24 | miRecords | Experimentally Observed |
|  | E2F5 | hsa-let-7f | TargetScan Human | High (predicted) |
|  | E2F6 | hsa-let-7f | TargetScan Human | High (predicted) |
|  | FANCD2 | hsa-let-7f | miRecords | Experimentally Observed |
|  | FAS | hsa-let-7f | TargetScan Human | High (predicted) |
|  | FASLG | hsa-let-7f | TargetScan Human | High (predicted) |
|  |  | hsa-miR-24 | TargetScan Human | High (predicted) |
|  | FZD3 | hsa-let-7f | TargetScan Human | High (predicted) |
|  | FZD4 | hsa-let-7f | TargetScan Human | High (predicted) |
|  | FZD5 | hsa-miR-24 | TargetScan Human | High (predicted) |
|  | GAB2 | hsa-let-7f | TargetScan Human | High (predicted) |
|  |  | hsa-miR-24 | TargetScan Human | High (predicted) |
|  | GNAL | hsa-let-7f | TargetScan Human | High (predicted) |
|  | GNAT1 | hsa-let-7f | TargetScan Human | High (predicted) |
|  | GSK3B | hsa-miR-24 | TargetScan Human | High (predicted) |
|  | HIPK2 | hsa-let-7f | TargetScan Human | High (predicted) |
|  | HRAS | hsa-let-7f | miRecords | Experimentally Observed |
|  | KRAS | hsa-let-7f | TarBase, miRecords | Experimentally Observed |
|  | MAP2K4 | hsa-miR-24 | miRecords | Experimentally Observed |
|  | MAPK11 | hsa-let-7f | TargetScan Human | High (predicted) |
|  | MAPK14 | hsa-miR-24 | TarBase, TargetScan Human, miRecords | Experimentally Observed, High (predicted) |
|  | MAPK9 | hsa-let-7f | TargetScan Human | High (predicted) |
|  | MYC | hsa-miR-24 | TargetScan Human, miRecords | Experimentally Observed, Moderate (predicted) |
|  |  | hsa-let-7f | Ingenuity Expert Findings, TarBase, miRecords | Experimentally Observed |
|  | NFKBIE | hsa-miR-24 | TargetScan Human | High (predicted) |
|  | NLK | hsa-miR-24 | TargetScan Human | High (predicted) |
|  |  | hsa-let-7f | TargetScan Human | High (predicted) |
|  | NOTCH1 | hsa-miR-24 | TarBase | Experimentally Observed |
|  | NRAS | hsa-let-7f | TargetScan Human, miRecords | Experimentally Observed, High (predicted) |
|  | PAK1 | hsa-let-7f | TargetScan Human | High (predicted) |
|  | PAK4 | hsa-miR-24 | TargetScan Human | High (predicted) |
|  | PIK3R3 | hsa-miR-24 | TargetScan Human | High (predicted) |
|  | PMAIP1 | hsa-let-7f | TargetScan Human | High (predicted) |
|  | PRKCA | hsa-miR-24 | TargetScan Human | High (predicted) |
|  | PRKCH | hsa-miR-24 | TargetScan Human | High (predicted) |
|  | PRKD3 | hsa-miR-24 | TargetScan Human | High (predicted) |
|  | RALA | hsa-miR-24 | TargetScan Human | High (predicted) |
|  | RALB | hsa-let-7f | TargetScan Human | High (predicted) |
|  | RAP1A | hsa-miR-24 | TargetScan Human | High (predicted) |
|  | RAP1B | hsa-miR-24 | TargetScan Human | High (predicted) |
|  | RAP2A | hsa-miR-24 | TargetScan Human | High (predicted) |
|  | RASA1 | hsa-miR-24 | TargetScan Human | High (predicted) |
|  | RASGRP1 | hsa-let-7f | TargetScan Human | High (predicted) |
|  | RB1 | hsa-let-7f | TargetScan Human | High (predicted) |
|  | RBL1 | hsa-miR-24 | TargetScan Human | High (predicted) |
|  | RHOB | hsa-let-7f | TarBase | Experimentally Observed |
|  | RHOG | hsa-let-7f | TarBase | Experimentally Observed |
|  | SMAD2 | hsa-let-7f | TargetScan Human | High (predicted) |
|  | SMAD3 | hsa-miR-24 | miRecords | Experimentally Observed |
|  | SMAD4 | hsa-miR-24 | miRecords | Experimentally Observed |
|  | SMAD5 | hsa-miR-24 | miRecords | Experimentally Observed |
|  | TAB2 | hsa-let-7f | TargetScan Human | High (predicted) |
|  | TGFBR1 | hsa-let-7f | TargetScan Human, miRecords | Experimentally Observed, High (predicted) |
|  | TP53 | hsa-let-7f | TargetScan Human | High (predicted) |
|  | WNT1 | hsa-let-7f | TargetScan Human, miRecords | Experimentally Observed, High (predicted) |
| II: Estrogen-mediated S-phase Entry | CCNA2 | hsa-miR-24 | miRecords | Experimentally Observed |
|  | CCND1 | hsa-let-7f | Ingenuity Expert Findings, TarBase, TargetScan Human, miRecords | Experimentally Observed, High (predicted) |
|  | CDC25A | hsa-let-7f | TargetScan Human, miRecords | Experimentally Observed, High (predicted) |
|  | CDK1 | hsa-miR-24 | miRecords | Experimentally Observed |
|  | CDK4 | hsa-miR-24 | miRecords | Experimentally Observed |
|  | CDKN1A | hsa-let-7f | TargetScan Human | High (predicted) |
|  | CDKN1B | hsa-miR-24 | TargetScan Human | High (predicted) |
|  | E2F2 | hsa-miR-24 | miRecords | Experimentally Observed |
|  |  | hsa-let-7f | TargetScan Human | High (predicted) |
|  | E2F5 | hsa-let-7f | TargetScan Human | High (predicted) |
|  | E2F6 | hsa-let-7f | TargetScan Human | High (predicted) |
|  | ESR2 | hsa-let-7f | TargetScan Human | High (predicted) |
|  | MYC | hsa-miR-24 | TargetScan Human, miRecords | Experimentally Observed,Moderate (predicted) |
|  |  | hsa-let-7f | Ingenuity Expert Findings, TarBase, miRecords | Experimentally Observed |
|  | RB1 | hsa-let-7f | TargetScan Human | High (predicted) |
|  | RBL1 | hsa-miR-24 | TargetScan Human | High (predicted) |
| III: PTEN Signaling | AKT2 | hsa-let-7f | TargetScan Human | High (predicted) |
|  | BCL2L1 | hsa-let-7f | Ingenuity Expert Findings, TargetScan Human | Experimentally Observed, High (predicted) |
|  | BCL2L11 | hsa-miR-24 | TargetScan Human | High (predicted) |
|  | BMPR1B | hsa-miR-24 | TargetScan Human | High (predicted) |
|  | BMPR2 | hsa-miR-24 | TargetScan Human | High (predicted) |
|  | CASP3 | hsa-let-7f | TargetScan Human, miRecords | Experimentally Observed, High (predicted) |
|  | CBL | hsa-let-7f | TargetScan Human | High (predicted) |
|  | CCND1 | hsa-let-7f | Ingenuity Expert Findings, TarBase, TargetScan Human, miRecords | Experimentally Observed, High (predicted) |
|  | CDKN1A | hsa-let-7f | TargetScan Human | High (predicted) |
|  | CDKN1B | hsa-miR-24 | TargetScan Human | High (predicted) |
|  | CHUK | hsa-let-7f | TargetScan Human | High (predicted) |
|  | FASLG | hsa-let-7f | TargetScan Human | High (predicted) |
|  |  | hsa-miR-24 | TargetScan Human | High (predicted) |
|  | FGFR3 | hsa-miR-24 | TargetScan Human | High (predicted) |
|  | GHR | hsa-let-7f | TargetScan Human | High (predicted) |
|  | GSK3B | hsa-miR-24 | TargetScan Human | High (predicted) |
|  | HRAS | hsa-let-7f | miRecords | Experimentally Observed |
|  | IGF1R | hsa-let-7f | TargetScan Human | High (predicted) |
|  | IKBKB | hsa-miR-24 | TargetScan Human | High (predicted) |
|  | IKBKE | hsa-let-7f | TargetScan Human | High (predicted) |
|  | INPP5B | hsa-miR-24 | TargetScan Human | High (predicted) |
|  | INSR | hsa-let-7f | TargetScan Human | High (predicted) |
|  | ITGA3 | hsa-miR-24 | TargetScan Human | High (predicted) |
|  | KRAS | hsa-let-7f | TarBase, miRecords | Experimentally Observed |
|  | MAGI1 | hsa-miR-24 | TargetScan Human | High (predicted) |
|  | NRAS | hsa-let-7f | TargetScan Human, miRecords | Experimentally Observed, High (predicted) |
|  | NTRK3 | hsa-let-7f | TargetScan Human | High (predicted) |
|  | PDGFRA | hsa-miR-24 | TargetScan Human | High (predicted) |
|  | PDGFRB | hsa-miR-24 | TargetScan Human | High (predicted) |
|  | PIK3R3 | hsa-miR-24 | TargetScan Human | High (predicted) |
|  | TGFBR1 | hsa-let-7f | TargetScan Human, miRecords | Experimentally Observed, High (predicted) |
|  | TGFBR3 | hsa-let-7f | TargetScan Human | High (predicted) |
| IV: Glioma Signaling | AKT2 | hsa-let-7f | TargetScan Human | High (predicted) |
|  | CALM1 (includes others) | hsa-let-7f | TargetScan Human | High (predicted) |
|  | CAMK2B | hsa-miR-24 | TargetScan Human | High (predicted) |
|  | CAMK2D | hsa-miR-24 | TargetScan Human | High (predicted) |
|  | CCND1 | hsa-let-7f | Ingenuity Expert Findings, TarBase, TargetScan Human, miRecords | Experimentally Observed, High (predicted) |
|  | CDK4 | hsa-miR-24 | miRecords | Experimentally Observed |
|  | CDK6 | hsa-let-7f | TargetScan Human, miRecords | Experimentally Observed, High (predicted) |
|  | CDKN1A | hsa-let-7f | TargetScan Human | High (predicted) |
|  | CDKN2A | hsa-miR-24 | TarBase, miRecords | Experimentally Observed |
|  | E2F2 | hsa-let-7f | TargetScan Human | High (predicted) |
|  |  | hsa-miR-24 | miRecords | Experimentally Observed |
|  | E2F5 | hsa-let-7f | TargetScan Human | High (predicted) |
|  | E2F6 | hsa-let-7f | TargetScan Human | High (predicted) |
|  | HRAS | hsa-let-7f | miRecords | Experimentally Observed |
|  | IGF1 | hsa-let-7f | TargetScan Human | High (predicted) |
|  | IGF1R | hsa-let-7f | TargetScan Human | High (predicted) |
|  | KRAS | hsa-let-7f | TarBase,miRecords | Experimentally Observed |
|  | NRAS | hsa-let-7f | TargetScan Human, miRecords | Experimentally Observed, High (predicted) |
|  | PDGFB | hsa-let-7f | TargetScan Human | High (predicted) |
|  | PDGFRA | hsa-miR-24 | TargetScan Human | High (predicted) |
|  | PDGFRB | hsa-miR-24 | TargetScan Human | High (predicted) |
|  | PIK3R3 | hsa-miR-24 | TargetScan Human | High (predicted) |
|  | PRKCA | hsa-miR-24 | TargetScan Human | High (predicted) |
|  | PRKCH | hsa-miR-24 | TargetScan Human | High (predicted) |
|  | PRKD3 | hsa-miR-24 | TargetScan Human | High (predicted) |
|  | RB1 | hsa-let-7f | TargetScan Human | High (predicted) |
|  | RBL1 | hsa-miR-24 | TargetScan Human | High (predicted) |
|  | TP53 | hsa-let-7f | TargetScan Human | High (predicted) |
| V: Chronic myeloid leukemia signaling | AKT2 | hsa-let-7f | TargetScan Human | High (predicted) |
|  | BCL2L1 | hsa-let-7f | Ingenuity Expert Findings, TargetScan Human | Experimentally Observed, High (predicted) |
|  | CCND1 | hsa-let-7f | Ingenuity Expert Findings, TarBase, TargetScan Human, miRecords | Experimentally Observed, High (predicted) |
|  | CDK4 | hsa-miR-24 | miRecords | Experimentally Observed |
|  | CDK6 | hsa-let-7f | TargetScan Human, miRecords | Experimentally Observed, High (predicted) |
|  | CDKN1A | hsa-let-7f | TargetScan Human | High (predicted) |
|  | CDKN1B | hsa-miR-24 | TargetScan Human | High (predicted) |
|  | CDKN2A | hsa-miR-24 | TarBase, miRecords | Experimentally Observed |
|  | CHUK | hsa-let-7f | TargetScan Human | High (predicted) |
|  | E2F2 | hsa-let-7f | TargetScan Human | High (predicted) |
|  |  | hsa-miR-24 | miRecords | Experimentally Observed |
|  | E2F5 | hsa-let-7f | TargetScan Human | High (predicted) |
|  | E2F6 | hsa-let-7f | TargetScan Human | High (predicted) |
|  | GAB2 | hsa-let-7f | TargetScan Human | High (predicted) |
|  |  | hsa-miR-24 | TargetScan Human | High (predicted) |
|  | HRAS | hsa-let-7f | miRecords | Experimentally Observed |
|  | IKBKB | hsa-miR-24 | TargetScan Human | High (predicted) |
|  | IKBKE | hsa-let-7f | TargetScan Human | High (predicted) |
|  | KRAS | hsa-let-7f | TarBase, miRecords | Experimentally Observed |
|  | MYC | hsa-let-7f | Ingenuity Expert Findings, TarBase, miRecords | Experimentally Observed |
|  |  | hsa-miR-24 | TargetScan Human, miRecords | Experimentally Observed, Moderate (predicted) |
|  | NRAS | hsa-let-7f | TargetScan Human, miRecords | Experimentally Observed, High (predicted) |
|  | PIK3R3 | hsa-miR-24 | TargetScan Human | High (predicted) |
|  | RB1 | hsa-let-7f | TargetScan Human | High (predicted) |
|  | RBL1 | hsa-miR-24 | TargetScan Human | High (predicted) |
|  | SMAD3 | hsa-miR-24 | miRecords | Experimentally Observed |
|  | SMAD4 | hsa-miR-24 | miRecords | Experimentally Observed |
|  | TGFBR1 | hsa-let-7f | TargetScan Human, miRecords | Experimentally Observed, High (predicted) |
|  | TP53 | hsa-let-7f | TargetScan Human | High (predicted) |
